# Polariton-assisted excitation energy channeling in organic heterojunctions

**DOI:** 10.1038/s41467-021-22183-3

**Published:** 2021-03-25

**Authors:** Mao Wang, Manuel Hertzog, Karl Börjesson

**Affiliations:** Department of Chemistry and Molecular Biology, University of Gothenburg, Gothenburg, Sweden

**Keywords:** Energy transfer, Excited states, Electronic and spintronic devices

## Abstract

Exciton-polaritons are hybrid light-matter states resulting from strong exciton-photon coupling. The wave function of the polariton is a mixture of light and matter, enabling long-range energy transfer between spatially separated chromophores. Moreover, their delocalized nature, inherited from the photon component, has been predicted to enhance exciton transport. Here, we strongly couple an organic heterojunction consisting of energy/electron donor and acceptor materials to the same cavity mode. Using time-resolved spectroscopy and optoelectrical characterization, we show that the rate of exciton harvesting is enhanced with one order of magnitude and the rate of energy transfer in the system is increased two- to threefold in the strong coupling regime. Our results exemplify two means of efficiently channeling excitation energy to a heterojunction interface, where charge separation can occur. This study opens a new door to increase the overall efficiency of light harvesting systems using the tool of strong light-matter interactions.

## Abstract (web summary)

Exploiting delocalized organic polaritons for enhanced exciton harvesting has been advantageous for organic optoelectronic with planar heterojunctions. Here, the authors report polariton-assisted excitation energy channeling in organic heterojunctions coupled to the same cavity mode.

## Introduction

Diffusion of excited state energy is a key process in both photosynthesis^1^ and in organic optoelectronic devices^2–6^. In organic heterojunction photovoltaic devices (OPVs), the formed excited states (excitons) must migrate to an interface, constituted by one electron donating and one electron accepting material, and then dissociate into free charge carriers. However, the diffusion length of excitons in organic materials is short (a few nanometers), compared to the optical path length (>50 nm), thus severely limiting the efficiency of planar heterojunction (HJ) systems^6^. In a bulk HJ, the electron donating and accepting materials are blended together, which reduces the average distance that the exciton need to migrate^7,8^. This configuration has achieved a huge success and nearly all state-of-the-art OPV devices adopt some form of bulk HJ^9,10^. However, a blended configuration introduces new complications. The precise control of the nanoscale morphology remains challenging, and although enormous efforts have been made, no general protocol can be followed to optimize various molecular systems^11,12^. Furthermore, the morphology of the blended structure is acquired by kinetic rather than thermodynamic means. Long-term stability will therefore always be reduced when adopting this strategy^13^. An alternative method to increase the overall efficiency of exciton harvesting is by increasing the exciton diffusion length. It has been shown that resonance energy transfer between the donor and acceptor materials can increase the effective diffusion to the interface^14–16^. The experimentally observed effect is modest, but simulations have shown that a long-range energy transfer mechanism would result in an efficient harvesting of excitation energy^17^. This would allow the construction of an efficient OPV with a simple layered structure.

Polaritons are quasiparticles formed when light and matter are strongly coupled together^18^. Organic polaritons are formed when a molecular transition is on resonance with an optical cavity and the exchange of energy between the two is faster than the energy dissipates from the system^19–24^. Polaritons are inherently delocalized with a delocalization volume covering the whole cavity, and when two different excitons couple simultaneously to the same resonant mode of an optical cavity, the length scale of energy transfer has been shown to dramatically increase compared to that of typical Förster resonance energy transfer distances (<10 nm)^25–31^. Moreover, organic polaritonic photodiodes and OCSs have recently been made^32,33^, and in-plane exciton transport at micrometer length scale in disordered organic materials in the strong coupling regime was recently observed^34,35^. Organic polaritons can increase the overall efficiency of exciton harvesting in planar HJs in two different ways, by their delocalized nature and by long-range energy transfer, thus potentially enabling efficient OPVs from simple planar HJ structures.

In this study, we demonstrate that the excitation energy is efficiently channeled to the HJ interface when both the donor and acceptor molecules are strongly coupled to the same cavity mode. Using time-resolved spectroscopy and photodiode characterization, we show that the effective rate of exciton harvesting in the system is enhanced by one order of magnitude in the strong coupling regime. Furthermore, the efficiency of energy transfer from the donor to the acceptor is approximately threefold increased. The results provide a new window to enhance the overall efficiency in light harvesting systems using strong light-matter coupling.

## Results

### Introducing the system

**Fig. 1:**
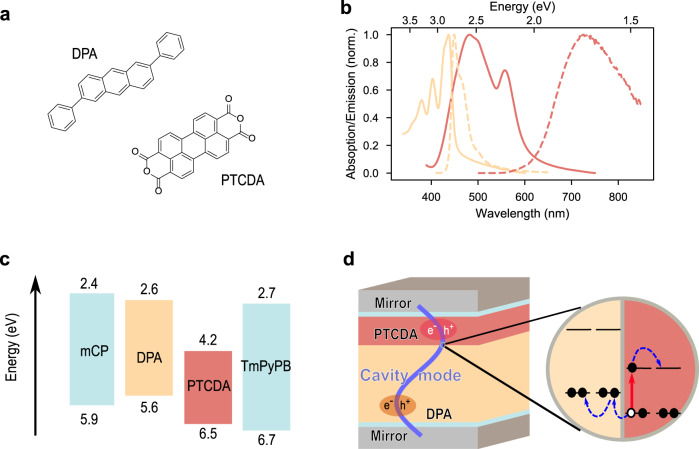
Molecular system and device architecture. **a** Molecular structures of 2,6-diphenylanthracene (DPA) and perylene-3,4,9,10-tetracarboxylic dianhydride (PTCDA). **b** The normalized absorption (solid line) and emission (dash line) spectra of DPA (yellow) and PTCDA (red) films. **c** Energy-level diagram of each molecule used in the study. To avoid any potential effect from the Ag mirrors, thin spacer layers was used. 10-nm mCP is chosen as the spacer between the bottom Ag mirror and DPA, and 15-nm TmPyPB between PTCDA and the top Ag mirror. The wide energy gap of the chosen materials enables them to act as an inert spacer, and neither energy nor electron transfer occurs between the spacers and active materials when in direct contact. **d** A typical structure of a Fabry–Pérot cavity in the study. From bottom to top: 30-nm Ag, 10-nm mCP, 163-nm DPA, 40-nm PTCDA, 15-nm TmPyPB, and 50-nm Ag. Schematics of the charge separation in the HJ are also shown.

To reach the strong coupling regime, molecular excitons need to interact strongly with a virtual photon inside a cavity. The strength of the light-matter coupling is proportional the transition dipole moment of the exciton being coupled, necessitating the use of highly absorbing molecules. Furthermore, to enable exciton dissociation at the donor–acceptor (D–A) interface, the energy levels of the two components should form a staggered gap, that is, a type II HJ. Here, 2,6-diphenylanthracene (DPA) and perylene-3,4,9,10-tetracarboxylic dianhydride (PTCDA) were used as energy/electron donor and acceptor materials, respectively (Fig. 1a)^36,37^. The two molecules have strong absorption properties (Fig. 1b), with thin film absorption coefficients of 4.8 × 10^4^ cm^−1^ (DPA) and 2.9 × 10^5^ cm^−1^ (PTCDA) at maximum position. More importantly, for the sake of spectroscopic analysis, the absorption spectra of the chosen molecules are well separated, enabling a clear demonstration of normal mode splitting in the strong coupling regime. The staggered energy levels of DPA and PTCDA (Fig. 1c) enable an efficient hole transfer from excited PTCDA to DPA, but the corresponding electron transfer from excited DPA to PTCDA is expected to be slower as compared to competing Förster type resonance energy transfer (details in Supplementary Note 1 and Supplementary Fig. 1). The schematic structure of the cavity system is shown in Fig. 1d, where DPA and PTCDA form a bilayer HJ incorporated in between two semitransparent Ag mirrors. To avoid any potential plasmonic effect from the Ag mirrors, thin spacer layers were used between the active layers and the Ag mirrors. As the absorption coefficient of the two molecules is different, the thickness of the DPA and PTCDA layers were chosen to keep their respective absorption abilities approximately equal. The structures of all layers in all cavities discussed in this study are summarized in the Supplementary Table 1.

### The strong coupling regime

**Fig. 2:**
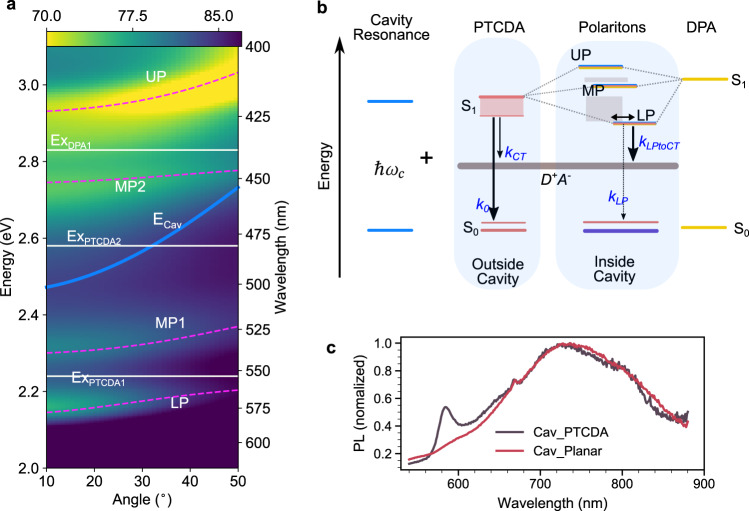
Angle-resolved reflectivity and Jablonski diagram of the system in the strong coupling regime. **a** Angle-resolved reflectivity (transverse electric mode) of a planar HJ cavity. The white solid lines indicate the excitons (Ex_PTCDA1_, Ex_PTCDA2_, and Ex_DPA1_) and the blue line the cavity energy (*E*_Cav_). The three excitons of PTCDA and DPA are strongly coupled to the $\lambda$ mode of the cavity, leading to the formation of four polaritonic modes (LP, MP1, MP2, and UP), indicated by magenta dashed lines, which are a fit to a coupled oscillator model. **b** Jablonski diagram of the system. In the diagram, the two excitons of PTCDA are represented by a broad S_1_ band to simplify the diagram without affecting the underlying physics. The cavity photon ($\hbar \omega _c$) couples to both PTCDA and DPA and by doing so form the upper (UP), middle (MP), and lower polaritons (LP). The gray-color bands represent dark states. k_0_ and k_CT_ are the decay rates of excited PTCDA to the ground state and charge-transfer state in the absence of a cavity, and k_LP_ and k_LPtoCT_ are the corresponding decay rates of LP to the ground state and charge-transfer states, respectively. The double arrow indicates the energy transfer between the exciton reservoir and LP. **c** Normalized emission spectra (excited at 475 nm) of the planar HJ cavity (Cav_Planar) and the reference cavity (Cav_PTCDA).

To investigate possible strong light-matter coupling in the cavity enclosed planar HJ, angle-resolved reflection spectroscopy was performed. DPA contributes to the light-matter coupling with its 0–0 transition, whereas PTCDA contributes with two transitions (Fig. 1b, spectroscopic analysis in Supplementary Note 2 and Supplementary Figs. 2–4). Figure 2a displays the angle-resolved reflectivity (TE mode) in which the molecular transitions and the cavity mode are marked with white and blue lines, respectively. A clear anticrossing behavior between the molecular transitions and the cavity mode is observed. The second mode of the cavity couples to the three molecular excitons of DPA and PTCDA, leading to the formation of four polariton branches (a full plot including the first cavity mode is shown in Supplementary Fig. 11). By fitting the data with a coupled oscillator model, the Rabi splittings were extracted. The obtained Rabi splittings are larger than the linewidths of the corresponding transitions (Supplementary Table 2), putting the system in the strong coupling regime. Worth noting, the formed polaritons are hybrid states having contributions from the donor, acceptor and cavity photon. This is especially true for the second middle polariton (MP2), which have roughly equal fractional contributions from the donor and acceptor molecules (Hopfield coefficients are shown in Supplementary Fig. 11 and Supplementary Table 3). The ratios of the PTCDA exciton, the DPA exciton, and the cavity photon in MP2 are 42%, 42%, and 14%, respectively. This middle polariton has been reported to play a key role in enhancing the energy transfer from high-energy polaritons and excitons to the lowest polariton^25,27,30^, a matter we will later develop further.

The photophysics of PTCDA in neat films has been extensively studied. The main absorption features consist of transitions to Frenkel (~2.54 eV) as well as mixed Frenkel charge-transfer states (~2.22 eV)^38^. Emission at room temperature arises from a low-energy excimeric state (~1.72 eV) having a very weak transition probability to the ground state (Supplementary Note 2)^38,39^. This low-energy transition arises from strong interchromophore coupling, and will therefore be affected if the high-energy Frenkel and charge-transfer states are perturbed. In our study, the high-energy transition is strongly coupled with the cavity, and the formed high-energy polariton is in turn coupled with the 0–0 transition (~2.85 eV) of DPA forming the upper and the MP2 polaritons. Two possible low-energy emission channels exist when considering excitations into the middle and lower polariton states: polariton emission, and emission from the low-energy excimeric state of PTCDA. We will consider both these emission channels when exploring the possibility of an increased channeling of excitation energy to the D–A interface in the strong coupling regime.

### Polariton-assisted exciton harvesting

**Fig. 3:**
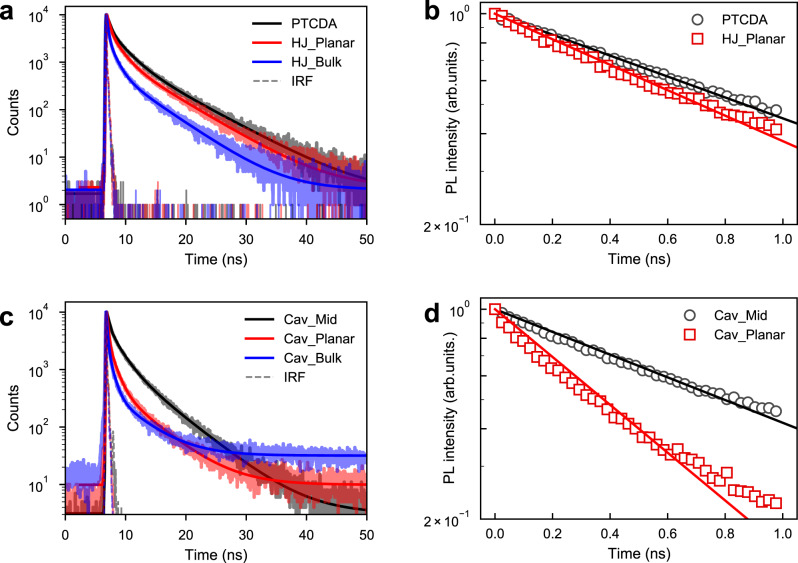
Time-resolved emission from HJ films and cavities. Time-resolved PL decay (shadow line) of films (**a**) and cavities (**c**) excited at 475 nm and probed at the emission position of PTCDA (720 nm), together with fitting to a three-exponential model (solid line). **b**, **d** The PL decays (scatter marker) and corresponding fittings (solid line) based on a rate-based model describing the dynamics of the energy relaxation in the systems.

Strong exciton–photon coupling leads to the formation of delocalized polaritonic states. Firstly, we will explore the possibility for these delocalized states to channel excited state energy to the interface of an organic HJ, and later we will explore if polariton-mediated long-range energy transfer can do the same. PTCDA in its excited state can donate an electron hole to an adjacent DPA molecule forming a charge-transfer state, which can be seen as a new channel of deactivating the excited state of PTCDA. This process can be studied by observing the effect of the presence of DPA on the PTCDA emission. Figure 2c displays emission from the planar HJ in a cavity and from a reference cavity only containing PTCDA. In the reference cavity, DPA is replaced by polystyrene (PS) to act as an inert layer and control the thickness of the cavity to ensure that PTCDA is coupled to same cavity mode (angular resolved reflectivity of all cavities is shown in Supplementary Figs. S11–16). Both cavities show emission from the low-energy transition of PTCDA at ~720 nm (1.72 eV). However, only the reference cavity displays polariton emission at ~580 nm (2.14 eV; Supplementary Fig. 12). The lower polariton constitutes of only PTCDA and the cavity mode in both cases (Hopfield coefficients in Supplementary Tables 3–8), which leads us to the conclusion that it is the DPA interface (which enables a deactivation route) that is responsible for the disappearance of polariton emission from the HJ. However, we cannot say from these data whether the deactivation route comes from the lower polariton or directly from the exciton reservoir, and because of overlapping emission from the low-energy PTCDA state, a quantification of rates using time-resolved spectroscopy would be difficult. Emission from the low-energy PTCDA state is however free of overlapping signals. The same decay channel responsible for outcompeting polariton emission, will also affect the low-energy PTCDA emission. Figure 3a displays the photoluminescence decay from PTCDA in presence and absence of an adjacent DPA layer. The average lifetime of PTCDA emission is shorter in the presence of a DPA layer (<τ_PTCDA_> = 3.73 ± 0.10 ns vs. <τ_PTCDA/DPA_> = 3.24 ± 0.10 ns, Table 1). Using a transfer matrix approach, we clarified that interference effects are not responsible for the difference in lifetimes (Supplementary Note 3 and Supplementary Figs. 5–8)^40,41^. Therefore, we attribute the reduction of the excited state lifetime of PTCDA to excitons diffusing and dissociating at the HJ interface.

**Table 1:** The lifetime of PTCDA and the rate of exciton harvesting toward the heterojunction interface in and outside cavities.

	Average lifetime of PTCDA (ns)^a^	Rate of exciton harvesting toward the CT states (×10^8^ s^−1^)
PTCDA	3.73 ± 0.10	–
Cav_PTCDA	3.78 ± 0.25	–
HJ_Planar	3.24 ± 0.10	1.73
Cav_Planar	1.97 ± 0.28	17.5
HJ_Bulk	2.29 ± 0.19	9.35

^a^The average lifetimes for all films and cavities are calculated based on a three-exponential model convoluted with the instrument response function (IRF): <*τ*> = $\mathop {\sum }\nolimits_i \tau _i^2B_i/(\tau _iB_i)$, where *B*_*i*_ is the amplitude of the corresponding component. The error bars of the average lifetimes are obtained from the deconvolution fitting of the decay.

Figure 3c displays the decays of the same PTCDA films, now enclosed in a cavity and verified to be within the strong coupling regime. The average lifetime of emission is 3.78 ± 0.25 and 1.97 ± 0.28 ns in the absence and presence of DPA, respectively. Cavity effects such as plasmonic effect from Ag mirrors, the Purcell effect, and the differences of the electric field and initial exciton distribution cannot explain this large reduction of the emission lifetime of PTCDA emission in presence of DPA in the strong coupling regime (Supplementary Note 3). Excitation-intensity dependent measurement shows no change of the lifetime from the HJ film and cavity, indicating no second-order effects present (Supplementary Fig. 6). Furthermore, when changing the thickness of the cavity in order to off tune the cavity and exciton energies completely, the lifetime of the PTCDA emission from the cavity is the same as it is outside the cavity (Supplementary Fig. 7), clearly showing that the enhanced quenching of PTCDA is an effect of strong exciton–photon coupling. Last but not the least, after inserting a thin polyvinyl alcohol (PVA) layer between DPA and PTCDA to remove the ability to form charge-transfer states while remaining in the strong coupling regime (Cav_Mid, cavity structure shown in Supplementary Fig. 15), the lifetime of PTCDA is the same as that of a bare film (Supplementary Fig. 7). Thus, it clearly demonstrates that the strong coupling regime enhances the ability of the HJ interface to quench the PTCDA excited state.

To quantify the exciton harvesting efficiency in the system, a classical rate-based model describing the dynamic processes was used. The interface of DPA–PTCDA opens up a new channel for deactivating excited PTCDA molecules. A rate equation can therefore be written in which the harvesting of excited state energy at the interface is modeled as an increased deactivation rate of the excited state

1 $$\frac{{d\left[ {{\mathrm{PTCDA}}^ \ast } \right]}}{{d\left[ t \right]}} = - k_0\left[ {{\mathrm{PTCDA}}^ \ast } \right] - k_{{\mathrm{CT}}}\left[ {{\mathrm{PTCDA}}^ \ast } \right]$$

where *k*_0_ is the sum of rates deactivating the excited state of PTCDA in absence of a DPA layer, and *k*_CT_ is the effective rate of exciton harvesting to interfacial charge-transfer states (a parameter that is also a function of PTCDA layer thickness). After a short excitation pulse, the intensity of emission is proportional to the concentration of PTCDA molecules in their excited state. The PL of PTCDA films does not follow a single exponential decay due to strong interactions between molecules and the disorder of molecular arrangement. However, in our system, the main effect of the quenching film (DPA) on the decay of PTCDA occurs at early times. The long emission tail almost keeps the same with and without DPA (Fig. 3). The initial decay of a neat PTCDA film follows a close to mono-exponential function, and was therefore used in the modeling. Using Eq. 1 (*k*_CT_ is equal to zero for this case), *k*_0_ in a neat film was determined to 8.0 × 10^8^ s^−1^ (Fig. 3b, black line). Furthermore, assuming that only one new decay pathway opens up in HJ_Planar compared to the neat PTCDA film, the rate of exciton harvesting (*k*_CT_) to the interface was determined to 1.7 × 10^8^ s^−1^ (Fig. 3b, red line).

In the strong coupling regime, polaritonic states form. The lower polaritonic state is the important one in the energy relaxation process when exciting PTCDA, and this therefore has to be included when modeling energy relaxation dynamics in the strong coupling regime^42^

2 $$\frac{{d\left[ {{\mathrm{PTCDA}}^ \ast } \right]}}{{d\left[ t \right]}} =	\; k_{{\mathrm{LP}}\,{\mathrm{to}}\,{\mathrm{PTCDA}}}\left[ {{\mathrm{LP}}} \right] - k_0\left[ {{\mathrm{PTCDA}}^ \ast } \right]\\ 	 - k_{{\mathrm{CT}}}\left[ {{\mathrm{PTCDA}}^ \ast } \right] - k_{{\mathrm{PTCDA}}\,{\mathrm{to}}\,{\mathrm{LP}}}\left[ {{\mathrm{PTCDA}}^ \ast } \right]$$

3 $$\frac{{d\left[ {{\mathrm{LP}}} \right]}}{{d\left[ t \right]}}\,=\,\, 	k_{{\mathrm{PTCDA}}\,{\mathrm{to}}\,{\mathrm{LP}}}\left[ {{\mathrm{PTCDA}}^ \ast } \right] - k_{{\mathrm{LP}}}\left[ {{\mathrm{LP}}} \right]\\ 	 - k_{{\mathrm{LP}}\,{\mathrm{to}}\,{\mathrm{CT}}}\left[ {{\mathrm{LP}}} \right] - k_{{\mathrm{LP}}\,{\mathrm{o}}\,{\mathrm{PTCDA}}}\left[ {{\mathrm{LP}}} \right]$$

where *k*_LP_ is the total rate of LP decay to the ground state in absence of a DPA/PTCDA interface, and *k*_LPtoCT_ is the rate of polariton-assisted exciton harvesting, which is the parameter that we are interested in. *k*_PTCDAtoLP_ (*k*_LPtoPTCDA_) refers to the rate of energy transfer from the exciton reservoir to the LP state (and vice versa). From previous reports, the energy relaxation from the exciton reservoir to LP is facilitated by: (1) the phonon scattering^43,44^ and (2) the radiative pumping from uncoupled molecules to LP^45^. The relaxation rate varies within a broad range of 10^9^–10^13^ s^−1^ depending on the molecule and cavity systems. In PTCDA cavities, the envelopes of polaritonic and PTCDA emission overlap to a large extend, and emission is predominantly PTCDA like (Fig. 2c). For such systems, the backward energy transfer from LP to the exciton reservoir can play a significant role^46,47^. A parameter scan covering *k*_PTCDAtoLP_ and *k*_LPtoPTCDA_ values in a broad range (between 10^9^ and 10^13^ s^−1^) was conducted in the modeling and the results from the full parameter scan can be found in Supplementary Fig. 10. For the convenience of the discussion that follows, the rate of energy transfer from LP to the exciton reservoir (and vice versa) was set to 10^12^ s^−1^. These values results in a conservative estimate of *k*_LPtoCT_ (Supplementary Fig. 10). To obtain *k*_LP_, a cavity with a thin PVA layer in between DPA and PTCDA (Cav_Mid, Supplementary Fig. 15) was prepared. It prevents charge-transfer states to form, while keeping approximately the same polariton composition as Cav_Planar. Thus, the composition of LP is roughly the same and the rate of decay in absence of a quenching layer should stay constant. Using Eq. 2 and Eq. 3 to model the decay of Cav_Mid (*k*_LPtoCT_ is equal to zero for this case), *k*_LP_ was determined to 9.4 × 10^8^ s^−1^. Furthermore, assuming that the only new decay pathway opening up in Cav_Planar compared to Cav_Mid is decay toward charge-transfer states, the rate of polariton-assisted exciton harvesting (*k*_LPtoCT_) was determined to 1.75 × 10^9^ s^−1^. This analysis indicates an order of magnitude increase in the rate of harvesting excitons toward interfacial charge-transfer states. We attribute this enhancement effect to the delocalized nature of polartionic states. Worth noting, the rate of polariton-assisted exciton harvesting in a planar HJ surpass that in the bulk one in the weak coupling regime (rates summarized in Table 1), indicating that optically driven mixing may be as effective as physically blending. The polaritons assisted exciton harvesting can also be viewed in terms of the enhanced apparent exciton diffusion in the strong coupling regime. Based on a one-dimensional diffusion model (details in Supplementary Note 4 and Supplementary Fig. 9), the apparent exciton diffusion length of PTCDA was calculated to increase by threefold in the strong coupling regime. To summarize, a reduced emission lifetime of the electron acceptor PTCDA in presence of the electron donor DPA when in the strong coupling regime was observed. This reduced emission lifetime was interpreted as an increased efficiency of exciton harvesting to the CT states at the HJ interface in the strong coupling regime.

### A polaritonic organic photodiode

**Fig. 4:**
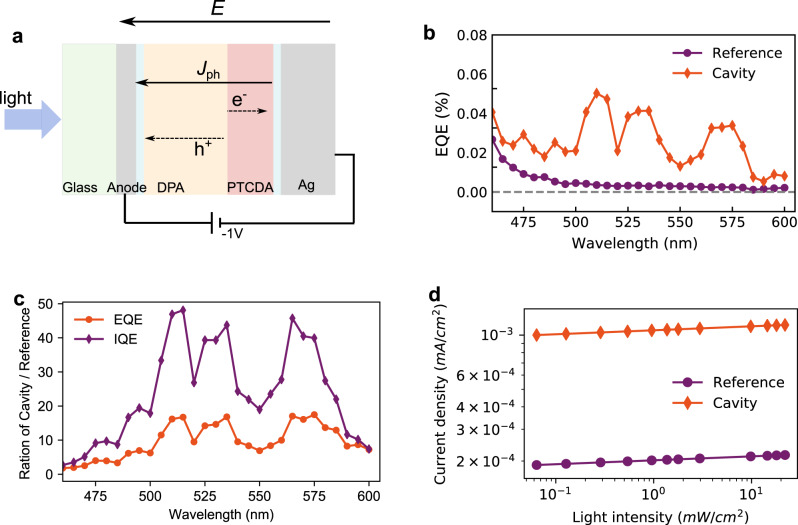
Photoresponse of the cavity and reference devices. **a** The device structures. The anode for the cavity device is 20 nm Ag and 110 nm ITO for the reference device. Spacer layers of mCP and TmPyPB were 5 and 10 nm, respectively. **b** The EQE of the photodiode under −1 V bias at short circuit condition. **c** The ratio of EQE and IQE of the cavity and reference devices. **d** The log–log plot of light intensity (475 nm) dependent photocurrent in cavity and reference devices.

To study the effect of polariton-assisted exciton harvesting on the overall photon-to-current efficiency, a cavity photodiode based on a planar HJ structure was fabricated. Device structures are shown in Fig. 4a and Supplementary Fig. 17. Two different devices were made, one containing a 20 nm Ag anode (cavity) and one containing a 110 nm ITO anode (reference). The structure was otherwise the same. The external quantum efficiency (EQE) of the two photodiodes is shown in Fig. 4b. As the donor/acceptor pair was chosen based on their spectroscopic properties and the cavity thickness were optimized for strong coupling, the EQE of both the cavity and the reference devices are low. However, the EQE of the cavity device is clearly higher than that of the reference. To further investigate the intrinsic photon-to-current efficiency, the internal quantum efficiencies (IQE) were calculated, which takes absorption differences of the two devices into account (Supplementary Fig. 17). Worth noting, significant amounts of molecules are uncoupled, especially at the nodes of the electromagnetic field. Furthermore, as the quantum efficiency in our reference device is very low, we thus assume that a reasonable large population of PTCDA molecules do not contribute significantly to the photocurrent. The consequence is the appearance of a “dip” in the EQE (and IQE) spectra at the position where the uncoupled molecules absorb (ca. 560 nm). The ratio of IQE (and EQE) between the cavity and reference devices is shown in Fig. 4c. Within the absorption range of PTCDA, the IQE (and EQE) of the cavity over reference devices is on average about 30 (12)-fold. Generally, the IQE depends on the following three steps: the efficiency of exciton diffusion to the interface ($\eta _{\mathrm{ED}}$), the charge separation at the interface ($\eta _{\mathrm{CS}}$), and the free charge extraction ($\eta _{\mathrm{CE}}$). As the charge-transfer state at the interface is uncoupled to the cavity, we assume that $\eta _{\mathrm{CS}}$ in both devices keep constant. To evaluate $\eta _{\mathrm{CE}}$, we conducted light intensity dependent photocurrent measurements. As the anode material is changed from Ag to ITO from cavity to reference, the dark current decreases (Supplementary Fig. 17). We attributed this to the increase of intrinsic resistance in the reference. However, as shown in Fig. 4d, the photocurrent (*J*_ph_) with increased illumination intensity (*P*) in both devices follow $J_{\mathrm{ph}} \propto P^\alpha$, where *α* is regarded as a measure of the efficiency of free charge extraction $( {\eta _{\mathrm{CE}}} )$ of the system^48,49^. The value of *α* obtained from the cavity and reference devices is similar, indicating that the change of anode material does not significantly affect $\eta _{\mathrm{CE}}$ in the two systems. Thus, the enhanced photon-to-current efficiency in the cavity device can be attributed to the increased efficiency of exciton harvesting to interfacial charge-transfer states $( {\eta _{\mathrm{ED}}} )$ in the strong coupling regime.

### Polariton-assisted energy transfer

We will now turn our attention to processes happening when the system is excited nonresonantly. In the strong coupling regime, the wave functions of the individual components mix to form the polaritonic states. This mixing of wave functions has been shown to effectively enable an ultrafast energy transfer event between the polariton states^27,30,50,51^. As shown in the Jablonski diagram in Fig. 2b, the main contributor to the upper polariton (UP) is DPA, and the UP is therefore mainly localized in the part of the cavity where DPA resides. After initial excitation of UP, the energy is relaxed to the middle polariton (MP2), which consist of roughly equal contributions from both molecular excitons. From there, the excited state energy is further relaxed to the lower polariton, which consists of contribution from PTCDA, and is therefore mainly localized in the part of the cavity where PTCDA resides. The relaxation to the lower polariton is going through the PTCDA exciton reservoirs and MP1, and through this scheme, the polaritonic states facilitates energy transfer from DPA to PTCDA.

**Fig. 5:**
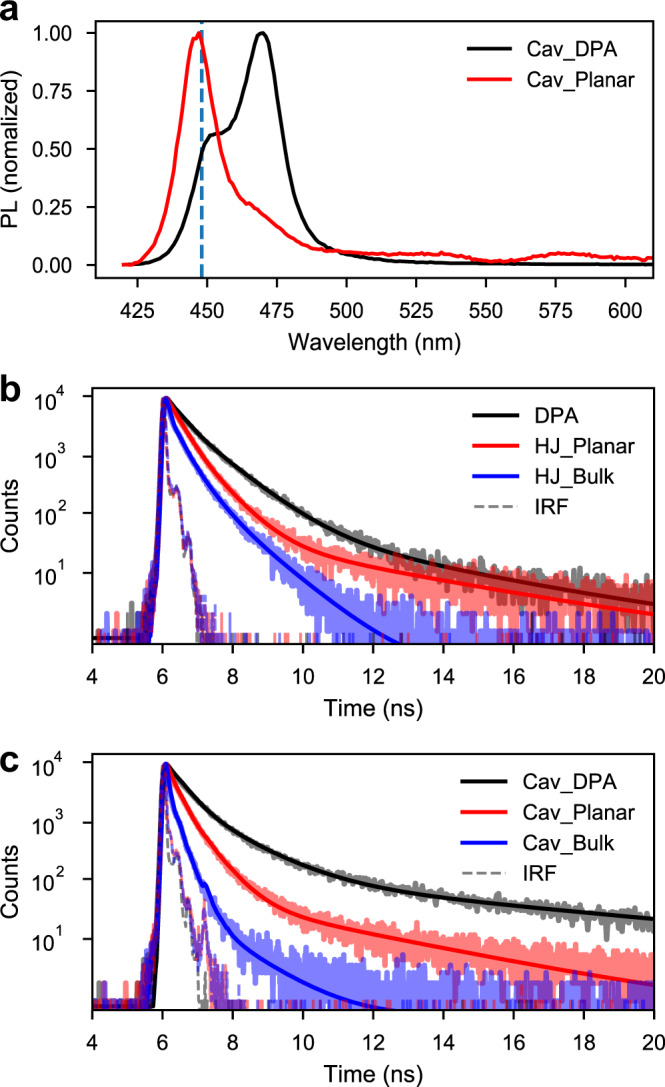
Steady-state and time-resolved emission from HJ films and cavities. **a** Steady-state emission from Cav_DPA (Ag/10 nm mCP/163 nm DPA/~80-nm PVA/Ag) and Cav_Planar HJs (Ag/10-nm mCP/163-nm DPA/40-nm PTCDA/15-nm TmPyPB/Ag) probed at normal angle. The wavelength of the excitation light was 405 nm, where only DPA absorbs. The vertical blue line (at 448 nm) indicates the emission from uncoupled DPA molecules. Time-resolved PL decay of films (**b**) and cavities (**c**) probed at the emission position of DPA (448 nm), together with fittings to a three-exponential model (solid line). The excitation wavelength was 405 nm. The black curves represent decays from DPA films, red curves represent planar HJs, and blue curves represent bulk HJs (10 nm mCP/163 nm DPA + 40 nm PTCDA/15 nm TmPyPB).

The incorporation of an efficient long-range energy transfer event in an organic HJ has been predicted to significantly increase the rate of charge separation^17^. Thus, by placing the HJ in the strong coupling regime, the polariton-assisted energy transfer is expected to increase the effective rate of exciton harvesting (hole transfer from PTCDA to DPA in our system). We can qualitatively study the effect of polariton-assisted energy transfer by observing the steady-state emission. Figure 5a shows the steady-state emission from cavities containing a HJ or a single layer of DPA when exciting at high energies nonresonantly (405 nm). To enable an equal contribution to the coupling strength of DPA between these two cavities, the thickness of the DPA layer is constant in both, and a PVA spacer layer was used in the single layer DPA cavity to tune it to resonance (Supplementary Fig. 14). In the single layer DPA cavity, strong coupling leads to the formation of the upper and lower polaritons. As shown in Fig. 5a, emission from the single layer DPA cavity shows both exciton emission (448 nm) as well as polariton emission (473 nm, dispersive emission spectra are shown in Supplementary Fig. 14). After addition of a PTCDA layer, only exciton emission from DPA is observed. The absence of polariton emission is indicative of an efficient energy transfer to lower energy states. The energy transfer event is facilitated by the MP2 state which is formed by the wave function mixing of DPA and PTCDA (42% DPA and 42% PTCDA, see Supplementary Table 3), followed by a relaxation to the PTCDA exciton reservoir. We propose that this energy relaxation pathway can be used to channel excitation energy (far from the interface) to PTCDA, from where hole transfer occurs.

In a strongly coupled exciton–photon system, the rate-limiting step is the decay from the exciton reservoir to the corresponding lower in energy polariton branch^43,52^. By comparing the rate of emission from the DPA exciton reservoir in presence and absence of the decay channel to PTCDA, the rate of polariton-assisted energy transfer can be calculated. Moreover, since a long-range energy transfer event results in an efficient charge transfer in a HJ^14,17^, most of the transferred energy is expected to result in a following hole transfer from PTCDA to DPA. Figure 5b, c displays time-resolved emission after nonresonant excitation from a single DPA layer, a HJ, and a bulk HJ, in absence and presence of a cavity, respectively. In the absence of a cavity (Fig. 5b), the average lifetime of a single DPA layer is 830 ps. When adding a layer of PTCDA the lifetime lowers to 560 ps, and for a bulk system the lifetime is further decreased to 340 ps. The reduction of the DPA lifetime in presence of a PTCDA layer is caused by FRET from DPA to PTCDA (Supplementary Note 1). The even shorter lifetime in the bulk geometry is due to a smaller average DPA–PTCDA distance in the bulk as compared to the layered structure. PTCDA provides an energy transfer based relaxation channel for DPA, and with this picture in mind, the average rate of energy transfer was calculated for the planar and bulk geometries to 0.6 × 10^9^ s^−1^ and 1.7 × 10^9^ s^−1^, respectively. In the presence of a cavity, the systems enter the strong coupling regime and now the average lifetime of a single layer of DPA is 1.64 ns. The lifetime monitored at the emission energy of the exciton (448 nm) and LP (473 nm) are the same, indicating that the rate-limiting step for the LP decay is the energy relaxation from the exciton reservoir to LP^43,50^. When adding a layer of PTCDA the lifetime reduces to 500 ps, which is further reduced to 150 ps if physically mixing the two molecules together. The average rates of energy transfer in cavities are calculated to be 1.4 × 10^9^ s^−1^ and 6.1 × 10^9^ s^−1^ for the planar and bulk systems, respectively. The average rates of energy transfer from DPA to PTCDA are thus 2–3 times larger in the strong coupling regime (see Supplementary Table 9). Strong coupling assisted energy transfer has previously been shown by others^25–27,53^; however, when putting it in the context of a HJ consisting of organic semiconductors, the polariton-assisted energy transfer results in a polariton-assisted exciton harvesting.

## Discussion

In summary, we have studied the emission decay dynamics of an organic HJ in the strong coupling regime and observe a decreased emission lifetime, which we interpret as an effective increased rate of exciton harvesting. The increase of overall quantum efficiency in a photodiode embedded cavity further validates the enhanced exciton harvesting in the strong coupling regime. The scope of the processes described could be viewed from the field of organic photovoltaics, in which the physical blending of donor and acceptor molecules are used to decrease distances and thus increase the yield of exciton harvesting. By coupling a planar HJ strongly to the vacuum field in an optical cavity, we find that the delocalized polaritons formed by wave function mixing of the donor, acceptor, and vacuum field increase the effective rates of exciton harvesting to charge-transfer states at the HJ. In the system under study, the optically driven mixing by the vacuum field is as efficient in channeling energy as the physical blending of the donor and acceptor molecules. These results demonstrate a new and promising method that enables energy migration on a length scale that is relevant to organic photovoltaics. This in a planar HJ, which is a much simpler and above all a thermodynamic low-energy structure in contrast to the widely adopted bulk HJ. Compared to photosynthesis in nature, where the energy harvested by antenna molecules is efficiently channeled to the collector in the reaction center by strong interactions between neighboring molecules, our system mimics the same process by mixing the wave functions of the molecules and the vacuum field, effectively channeling the excitation energy toward the reactive donor/acceptor interface. Although our system is not designed to efficiently extract charges from the charge-separated state, we believe it is possible to use the concept in other organic systems to achieve an enhanced overall efficiency of photon-to-charge/current using strong light-matter coupling.

## Methods

### Materials

DPA (CAS: 95950-70-2, >98.0% (sublimed)) was purchased from TCI Chemicals and used as received without further purification. PTCDA (CAS: 128-69-8, 97%), 1,3-Bis(N-carbazolyl)benzene (mCP, CAS: 550378-78-4, 97%), 1,3,5-Tri(m-pyridin-3-ylphenyl)benzene (TmPyPB, CAS: 921205-03-0, 99% (HPLC)), PS (CAS: 9003-53-6, average Mw ~280,000), and PVA (CAS: 9002-89-5, Mw 85,000–124,000, 99+%) were purchased from Sigma Aldrich and used as received. Prepatterned 8 pixel ITO substrates with a sheet resistance of 20 Ω/square were purchased from Ossila Ltd.

### Cavity and device fabrication

Ag mirrors of the Fabry–Perot cavities were fabricated by vacuum sputtering deposition (HEX, Korvus Technology). A semitransparent Ag film was sputtered on top of the glass substrate as the bottom mirror. The layers of the spacer molecules and organic semiconductors were thermally evaporated sequentially at high vacuum (base pressure of <5 × 10^−6^ mbar, HEX, Korvus Technologies) on the Ag mirror or glass substrates. All the HJ films and the corresponding cavities were prepared with the same batch of evaporation to ensure the same quality and thickness of the films. The bulk films were prepared by coevaporation of PTCDA (~0.3 Å/s) and DPA (~1.2 Å/s) at different rates to keep the ratio of PTCDA and DPA the same as it in the planar structures. PS was used to replace the DPA layer in the reference cavity—Cav_PTCDA. PS (40 mg mL^−1^ in toluene) was prepared by spin-coating (Laurell Technologies WS-650) at the spin-speed of 4500 r.p.m. PVA (1 mg mL^−1^ in Milli-Q water) used in the Cav_DPA was spin-coated at a spin-speed of 1000 r.p.m. Substrates with polymer layers were firstly annealed at 80 °C on a hot plate for 5 min, and kept in the high-vacuum deposition chamber overnight before deposition the next layer. A semitransparent 50 nm Ag mirror was sputtered on top of the organic layers to complete the cavities. For the photodiode, the top 120 nm Ag mirror was prepared by electron-beam evaporator.

### Optical and optoelectronic measurements

The refractive index of the small molecule organic semiconductors was measured using an ellipsometer (J.A. Woollam M2000). Steady-state absorption spectra were measured using a standard spectrophotometer (Lambda 950, Perkin Elmer). The angle-resolved reflectivity was measured using the same spectrophotometer equipped with the universal reflectance accessory—LAMBDA. Emission and excitation measurements were performed using an FLS1000 spectrofluorometer (Edinburgh Instruments). The angle-dependent steady-state emission was measured using a homemade goniometer consisting of two liquid light guides connected to the spectrofluorometer. The light from the fiber guiding the excitation light was focused on the sample at a fixed angle of 30°, and the emitted light was focused on the entrance of the second fiber, which was placed at different angles and was used to guide the light back to the detector of the spectrofluorometer. Fluorescence lifetime measurements were determined with a time-correlated single-photon counting system (FLS1000) using picosecond-pulsed diode lasers. The frequency of the pulsed laser was set as 5 MHz, with a pulse duration of 100 ps and fluence of ~0.5 nJ/cm^2^. Depending on the molecules to be studied, different sides of the cavity were excited. For the study on the dynamics of PTCDA exciton, the excitation light (475.0 ± 4.5 nm) was illuminated through the top mirror of the cavity and the emission was collected from the same side. For the study on energy transfer, the excitation light (405.0 ± 2.0 nm) passes through the glass substrate and illuminates through the bottom mirror, and the emission was collected from the same side. The samples was installed on an oblique sample holder to avoid reflection light. The angles of excitation and emission are ~52° respective to the normal line of the sample surface. All experiments were conducted at ambient condition.

Current–voltage characteristics and EQE measurement were measured using a Keithley 2636B measurement unit under light from a Xe lamp, which was coupled into a monochromator. The light intensities (*P*) were calibrated with a Si photodiode. The incident angle of illuminated light was kept at 0°. The size of devices was determined by the intercross of bottom and top electrodes (0.2 cm × 0.15 cm). Two cavity devices and two reference devices were examined with reproducible results. All devices were encapsulated with a light-curable epoxy (Ossila) in glovebox environment before measurements. The EQE was calculated by $\mathrm{EQE} = R \times 1240/\lambda \times 100\%$, where *R* is the light responsivity, $R = (J_{\mathrm{ph}} - J_{\mathrm{dark}})/P$, and *P* is the power of incident light.

### Coupled oscillator model

The observed polariton branches in the planar HJ cavity (Cav_Planar) were fitted with a 4-by-4 coupled oscillator model

$$	\left( {\begin{array}{*{20}{c}} {E_{\mathrm{Cav}} - i\hbar {{\Gamma }}_{\mathrm{Cav}}} & {V_1} & {V_2} & {V_3} \\ {V_1} & {Ex_{\mathrm{PTCDA1}} - i\hbar {{\Gamma }}_{x1}} & 0 & 0 \\ {V_2} & 0 & {Ex_{\mathrm{PTCDA2}} - i\hbar {{\Gamma }}_{x2}} & 0 \\ {V_3} & 0 & 0 & {Ex_{\mathrm{DPA1}} - i\hbar {{\Gamma }}_{x3}} \end{array}} \right)\\ 	 \left( {\begin{array}{*{20}{c}} \alpha \\ \beta \\ \gamma \\ \delta \end{array}} \right) = E\left( {\begin{array}{*{20}{c}} \alpha \\ \beta \\ \gamma \\ \delta \end{array}} \right)$$

where *α*, *β*, *γ*, and *δ* are the mixing coefficients of the eigenvectors of the strongly coupled systems. The Hopfield coefficients in each polariton are calculated as |*α*|^2^, |*β*|^*2*^, |*γ*|^2^, and |*δ*|^2^. *E*_Cav_ is the energy of cavity mode with the FWHM $\hbar {{\Gamma }}_{\mathrm{Cav}}$, and *Ex*_PTCDA1_, *Ex*_PTCDA2_, and *Ex*_DPA1_ the exciton energies with their FWHM $\hbar {{\Gamma }}_{x1}$, $\hbar {{\Gamma }}_{x2}$, and $\hbar {{\Gamma }}_{x3}$. *E* represents the eigenvalues corresponding to the energy of the formed polaritons. The cavity mode was determined as $E_{\mathrm{Cav}}\left( \theta \right) = E_0/\sqrt {1 - (\sin \theta /n_{\mathrm{eff}})^2}$. Here, *θ* is the angle of incidence and *E*_0_ is the cavity energy at *θ* = 0°. As the excitons are coupled to the second mode of the cavity, the cavity mode (*E*_0_) was obtained from the first mode of the cavity ($E_{0\_2^{nd}} = 2E_{0\_1^{st}}$). *n*_eff_ is the effective refractive index. The coupling potential *V* is related to the Rabi splitting $\hbar {{\Omega }}$, when *E*_Cav_ = *Ex* it is given by $2V = \sqrt {\hbar {{\Omega }}^2 + (\hbar {{\Gamma }}_{\mathrm{Cav}} - \hbar {{\Gamma }}_x)^2}$. Here, *V*_1_, *V*_2_, *V*_3_, and *n*_eff_ are obtained by fitting experimental data to the eigenvalues of the 4-by-4 matrix with the Levenberg–Marquardt method.

## Supplementary information

Supplementary Information

## Data Availability

The data that support the findings of this study are available from the corresponding author upon reasonable request.
