# Supplementary material for: Polariton-assisted excitation energy channeling in organic heterojunctions
Source: Nat Commun. 2021 Mar 25;12:1874. doi: 10.1038/s41467-021-22183-3 (PMC7994571; doi:10.1038/s41467-021-22183-3)
Supplement: Supplementary file 1 — Supplementary Information [file 41467_2021_22183_MOESM1_ESM.pdf]

Supplementary Information

Polariton-assisted excitation energy channeling in organic heterojunctions

*Mao Wang, Manuel Hertzog and Karl Börjesson**

University of Gothenburg, Department of Chemistry and Molecular Biology, Kemigården 4, 412 96 Gothenburg, Sweden.

E-mail: karl.borjesson@gu.se

Contents

Supplementary Note 1: Estimation of the energy and electron transfer rate S2

Supplementary Note 2: Optical properties of PTCDA thin film S4

Supplementary Note 3: Other potential reasons for the lifetime difference S7

Supplementary Note 4: Calculation of the exciton diffusion length S11

Supplementary Figure 10: Calculation of k_{LPtoCT} within a parameter scan S12

Supplementary Figure 11–Figure 16: Angle resolved reflectivity of all cavities S13

Supplementary Figure 17: The polaritonic photodiode S20

Supplementary Table 1: Cavity structures and the corresponding abbreviations S21

Supplementary Table 2: Rabi splittings and effective refractive indices S22

Supplementary Table 3-8: Hopfield coefficients of all cavities S22

Supplementary Table 9: Lifetime of DPA and the rate of energy transfer S24

Supplementary References S25

Supplementary Note 1: Estimation of the energy and electron transfer rate from DPA to PTCDA

There are two possible channels for the excitation relaxation when considering an excited DPA molecule and a ground state PTCDA molecule. (1) Förster-type resonance energy transfer (FRET) from DPA to PTCDA, which depends on the spectral overlap between the emission of DPA and the absorption of PTCDA (see Supplementary Figure 1a). (2) Electron transfer from the LUMO of DPA to the LUMO of PTCDA driven by the energy difference, ΔE_{LUMO}. A schematic indicating the two channels is shown in Supplementary Figure 2b inset.

When estimating the rate of FRET we follow the method proposed by McGehee *et al* to analyze the rate of FRET close to an interface.[1] The rate of energy transfer ($k_F(x)$) from an excited chromophore to a 3D semi-infinite array varies as $1/x^3$:

$$k_F(x) = \frac{C_A}{\tau}\frac{\pi}{6}\frac{R_0^6}{x^3} \quad (1)$$

where *x* is the distance to the planar interface, C_A is the density of acceptor chromophores (2.6 molecules nm^{-3}), R_0 is the Förster radius[2] calculated to 2.9 nm using Supplementary Equation 2:[3]

$$R_0^6 = 8.79 \times 10^{-5} * \frac{(\kappa^2 * Q_D)}{n^4} * \int_0^{\infty} F_D(\lambda) * \varepsilon_A(\lambda) * \lambda^4 \, \mathrm{d}\lambda \quad (2)$$

where κ^2 is the orientation factor (set to 2/3, random orientation is thus assumed), Q_D (7.0%) is the quantum yield of the DPA film measured using an integrated sphere, *n (1.70)* is the in-plane refractive index of the DPA film as measured by ellipsometry, F_D is the normalized spectral distribution of DPA fluorescence, and ε_A is the molar extinction coefficient of PTCDA. The calculated dependence of the rate of FRET (using Supplementary Equation 1) on the distance to the heterojunction is shown in Supplementary Figure 1b.

The non-adiabatic Marcus theory is the classical method to estimate the rate of electron transfer at a donor-acceptor heterojunction.[4,5] The rate of electron transfer (k_{ET}) is mainly decided by the offset energy (ΔE) and the reorganization energy (λ):

$$k_{ET} = \left(\frac{4\pi^3}{h^2 \lambda k_B T}\right)^{\frac{1}{2}} V^2 \exp\left(-\frac{(-\Delta E + \lambda)^2}{4\lambda k_B T}\right) \quad (3)$$

where *V* is the electronic coupling matrix element between the two states, *h* is Planck's constant, k_B is the Boltzmann constant and T is the temperature. However, it is challenging to calculate the electron transfer rate from this equation for complicated systems such as organic heterojunctions. Forrest *et al*. have investigated a large family of typical donor and acceptor combinations, and the results provide a reference to estimate the electron transfer rate in organic heterojunctions using the offset energy as an input.[5] The offset energy (ΔE_{LUMO}) for excited state electron transfer from DPA to PTCDA is 1.6~1.7

eV, which lies in the Marcus "inverted" region, giving an estimated rate of electron transfer in the interval 10^{10} to 10^{8} s^{-1} at the interface.

As charge transfer occurs only at the interface of the donor and acceptor, it should be compared to the estimated rate of FRET at the interface (within 2 nm), which is on the order of 10^{11} to 10^{13} s^{-1}. The rate of energy transfer is two order of magnitude faster than the estimated rate of electron transfer (see Supplementary Figure 1b) Thus, we conclude that FRET is the dominant relaxation pathway from excited DPA to PTCDA. Considering the charge transfer from excited PTCDA to DPA (hole transfer), the estimated rate is in the order of 10^{10} ~10^{11} s^{-1} based on the offset energy ($\Delta E_{HOMO} = 0.9\sim1.0\ eV$). It is at least one order of magnitude faster than the decay rate of the PTCDA exciton (on the order of 10^{8} ~10^{9} s^{-1}). The exciton dissociation efficiency at the interface can therefore be assumed to be unity.

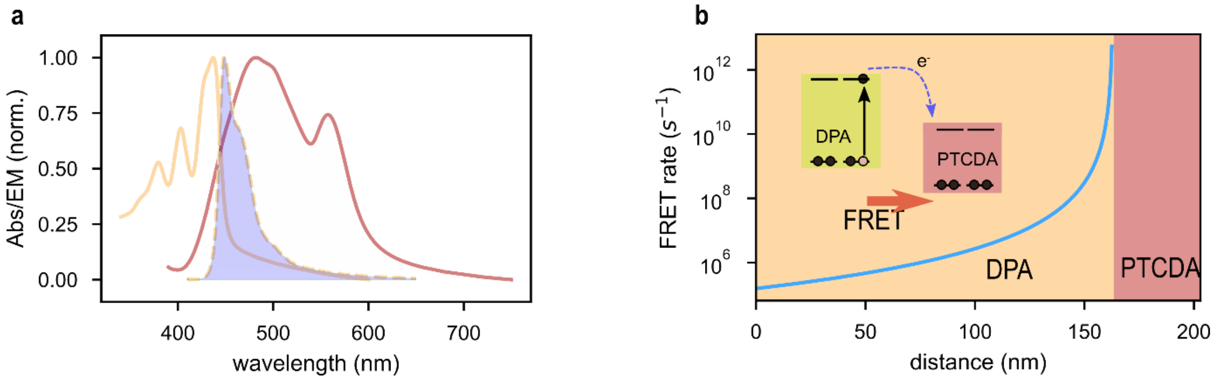

Supplementary Figure 1. **a** The spectral overlap (light blue area) of DPA's emission (dash yellow line) and the absorption of PTCDA (solid red line). **b** The dependence of the calculated rate of FRET (solid blue line) in the heterojunction. The thickness of DPA is 163 nm, and PTCDA 40 nm. At the interface (within 2 nm), the rate of FRET is on the order of 10^{11} to 10^{13} s^{-1}, much faster than the estimated rate of electron transfer ($k_{ET} \leq 10^{10}\ s^{-1}$). Inset: a schematic showing the two competing relaxation channels of excited DPA.

Supplementary Note 2: Optical properties of PTCDA thin film

PTCDA has been extensively studied due to its promising properties in applications such as optoelectronic devices. Thin PTCDA films prepared by vacuum deposition are known to crystallize into two polymorphic forms, α and β, depending on the growth conditions.[6] Here the thin-film X-ray diffraction of the films used in our experiments show a peak at 27.8º (see Supplementary Figure 2), indicating the existence of only the α-form.[7] It agrees with recent studies showing that films grown on quartz substrates at the room temperature consist only of the α-form.[8] The absorption and emission spectra of PTCDA are shown in Supplementary Figure 3b. The absorption profile of the PTCDA film is a combined effect of multiple species including Frenkel exciton, charge-transfer state, self-trapped charge-transfer state and a mixture of them. The interpretation of the precise nature of these species in the solid state of PTCDA is still under debate.[9,10] But it is generally accepted that the electronic absorption spectrum of PTCDA originates from both Frenkel and charge transfer transitions.[11] The main broad absorption being of Frenkel type and the low energy transition having a mix of Frenkel and charge transfer contributions (Supplementary Figure 3b).[12,13]

Emission from PTCDA at room temperature occurs at 1.72 eV (720 nm) and originates from an excimeric state.[14-16] This excimeric state has a very low transition dipole moment, giving a low rate of emission and negligible absorption. The PL spectrum is independent on the excitation photon energy, indicating an efficient relaxation of the excited state population to the lowest energy state. The Jablonski diagram for PTCDA is shown in Supplementary Figure 3a,[17] which is a complement to the simplified version in the main text (Figure 2b).

It requires multi components convolution to accurately describe the absorption spectrum of PTCDA because of the complicated photophysics in PTCDA films as mentioned above. The multi Frenkel excitons (FE state, Supplementary Figure 3a) have strong interactions between each other and can therefore be treated as one effective exciton in PTCDA thin films. Thus, the contribution from PTCDA to polaritons in the strong coupling regime is thus mainly from the Frenkel states (FE state, having a large oscillator strength) and the mixed charge-transfer Frenkel states (Mixed CT-FE state). A large transition dipole moment is the prerequisite to reach strong coupling regime. Although not completely following the profile of the absorption spectrum of PTCDA, a two-component deconvolution captures the main optical features of the PTCDA film (see Supplementary Figure 3a). Two excitons of PTCDA are therefore taken into account when considering the interaction with cavity photons.

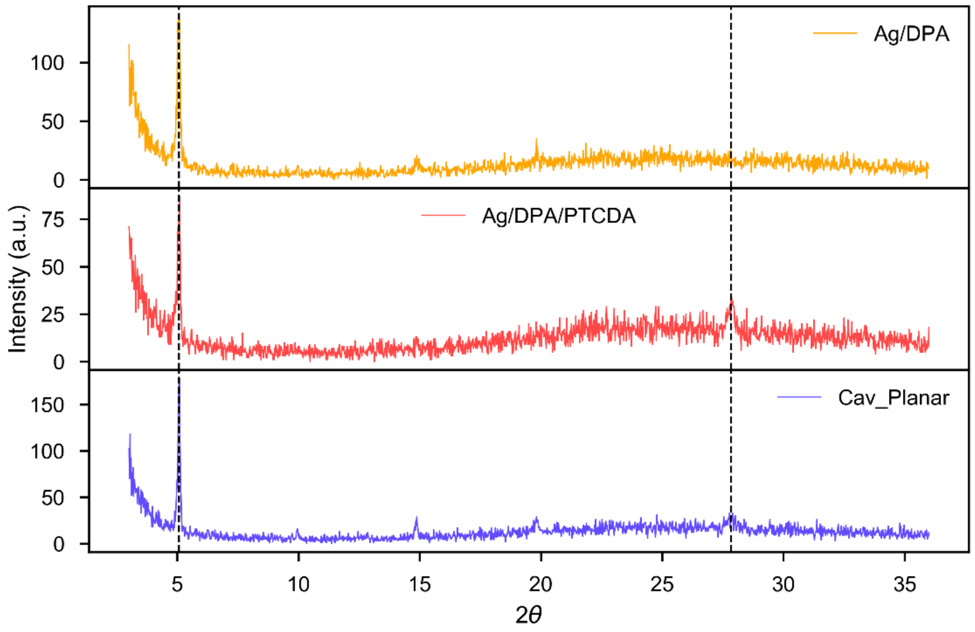

Supplementary Figure 2 The thin-film X-ray diffraction of samples studied in this paper. The peak at 5.1º is assigned to the DPA film, and the peak at 27.8º is assigned as the PTCDA film.

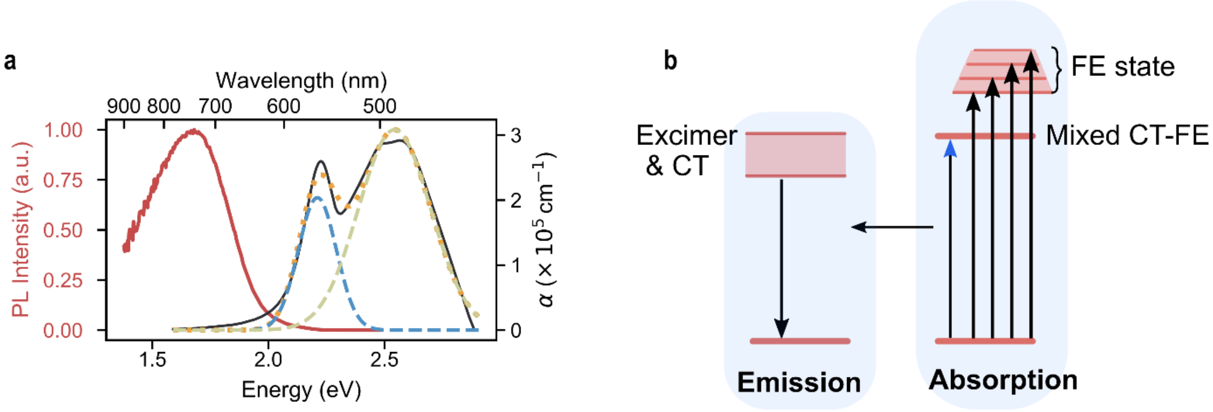

Supplementary Figure 3 a The photoluminescence (solid red line) and absorption spectra (solid dark line) of a 40-nm PTCDA film. A two-component deconvolution of the absorption spectrum is shown, in which the broad peak at high energy (dash grey line) consists of excitations to several vibronic levels. **b** The Jablonski diagram describing the energy states in a PTCDA film (at room temperature). The absorption of PTCDA is mainly attributed to the excitation to the multi Frenkel states (FE state) and a mixed charge-transfer Frenkel state (CT-FE). The emission at the room temperature is mainly from an excimeric state at relative low energy.

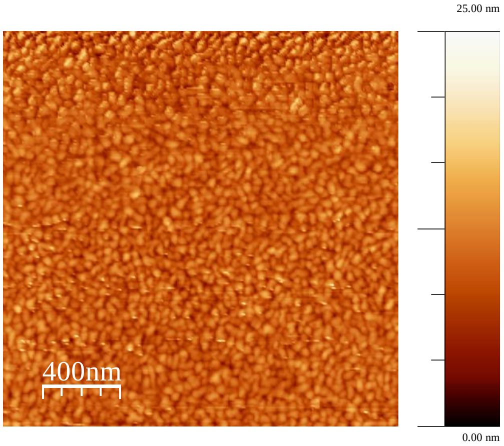

Supplementary Figure 4: AFM image of 40 nm PTCDA on Glass. It shows certain degree of crystallinity; in agree with the XRD results in Supplementary Figure 2. The grain size <*R*> is estimated to be 40 ± 15 nm. The root-mean-square surface roughness values is 2.7 nm.

Supplementary Note 3: Other potential reasons for the lifetime difference of samples in this study.

Electric field: The distribution of the electric field intensity inside samples depend on the surrounding dielectric environment. The electric field intensity can affect the emission properties of organic films, known as the effect of optical interference. This effect is obvious when there is a large difference of refractive indices between the quenching and non-quenching samples.[18] To explore the possibility of this effect in our system we calculated the distribution of the electric field intensity in all samples by the transfer matrix approach. For the reference PTCDA film and the PTCDA/DPA heterojunction, the distribution of the electric field intensities are shown in Supplementary Figure 5. As the refractive index of PS (1.61 at 475 nm) and DPA (1.73 at 475 nm) are quite close, the distribution of the electric field intensities are almost the same. Thus, the difference in the rate of emission between the PTCDA film and the planar HJ cannot be attributed to differences in the electric field.

Plasmonic effects of the Ag mirror: As both the reference and heterojunction films are enclosed in the cavity, the silver quenching effect is already compensated. Furthermore, the spacer layer (15-nm TmPyPB) avoids the potential damage of the active layer during sputtering of the top mirror. It also prevent the direct contact between PTCDA and the top silver mirror, and avoid the potential quenching effects of PTCDA from silver. Thus, the difference in the rate of emission between the reference cavity (Cav_PTCDA) and the HJ cavity (Cav_Planar) can not be attributed to the plasmonic effects of the silver mirror.

Purcell's effect: The Purcell effect is the enhancement of a quantum system's spontaneous rate of emission by its environment. In our study, the lifetime of the reference PTCDA film in and outside of cavity is the same (3.7±0.25 ns, see Table 1 in the main text). It indicates that the Purcell effect caused by the cavity here is negligible. The Purcell effect can be interpreted to be caused by a change of the density of final states during the emission process. As the optical density at 720 nm between the reference and planar cavity is almost the same (Supplementary Figure 5), the Purcell effect caused by the difference of the density of states is negligible. Thus, the difference in the rate of emission between reference cavity and the HJ cavity cannot be attributed to the Purcell effect.

Non-linear effects: Non-linear phenomena such as exciton-exciton annihilation can act as an additional decay channel, reducing the excited state lifetime of a material. To exclude any such processes, the time resolved PL of heterojunction films and cavities was recorded at different excitation dependencies. No effect of the lifetime with excitation power was observed (Supplementary Figure 6). Thus, the difference in the rate of emission between the reference cavity and the HJ cavity cannot be attributed to second order effects.

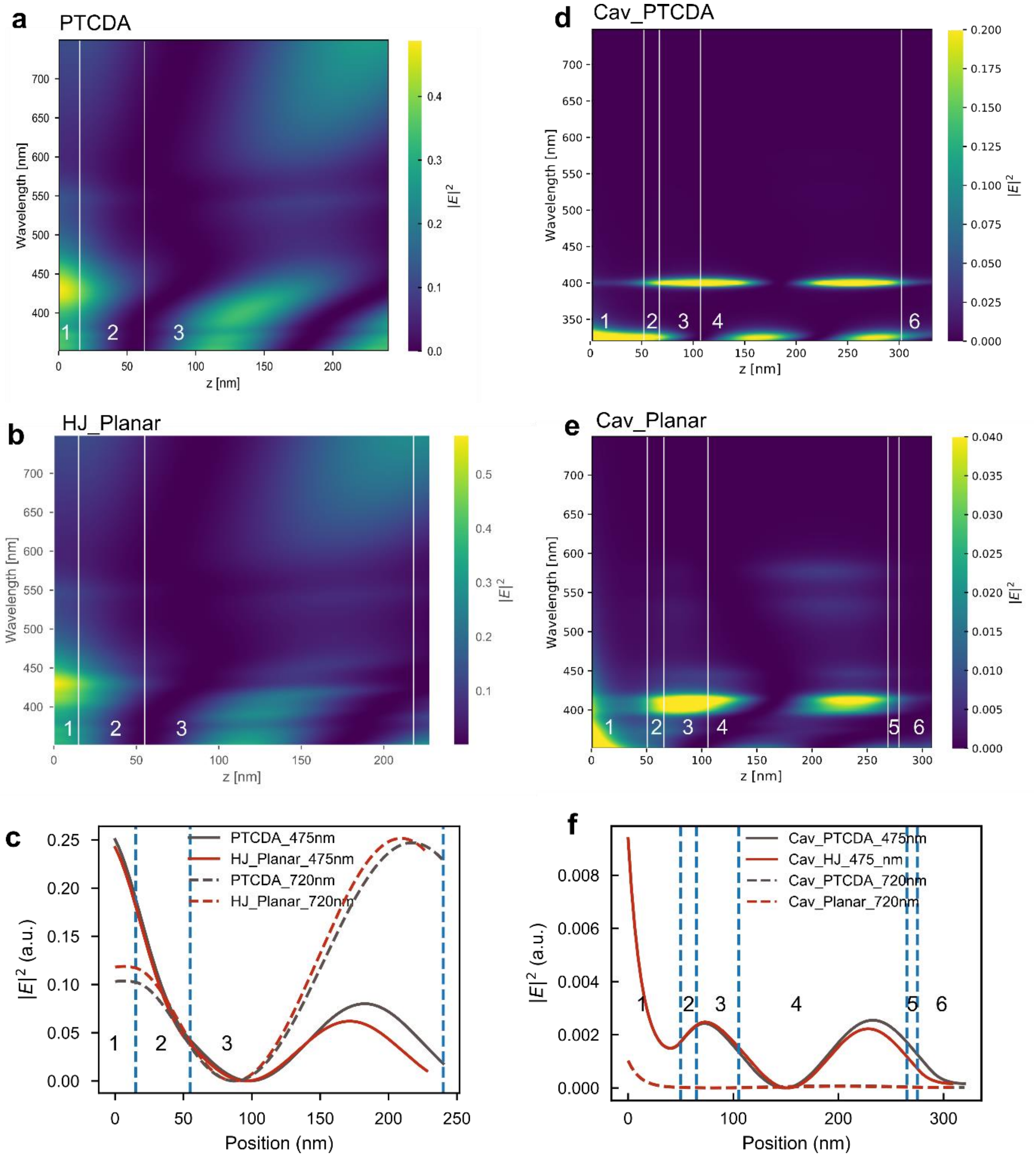

Supplementary Figure 5: The electric field distribution in PTCDA film (**a**), planar HJ (**b**), Cav_PTCDA (**d**) and Cav_Planar (**e**) as calculated by a transfer matrix approach. The comparison of electric field at 475 and 720 nm of film samples (**c**) and cavity samples (**f**). The vertical lines indicate the interface between different layers. In **a**, **b** and **c**, the sample structure is 15-nm TmPyPB (1), 40-nm PTCDA (2) and 163-nm DPA or 185-nm PS (3). In **d**, **e** and **f,** the sample structure is 50-nm Ag (1), 15-nm TmPyPB (2), 40-nm PTCDA (3), 163-nm DPA or 185-nm PS (4), 10-nm mCP (5) and 30-nm Ag (6).

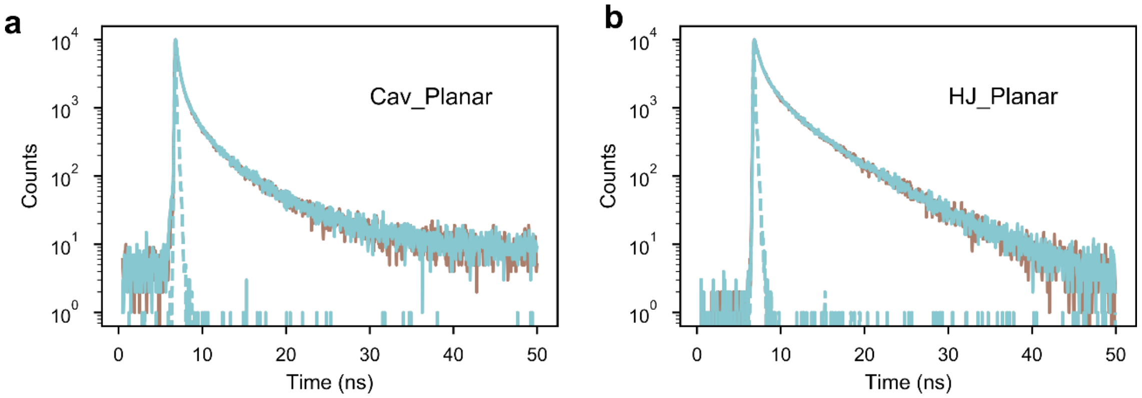

Supplementary Figure 6: The PL decay at 720nm (1.72eV) from a planar heterojunction cavity (a) and a film (b) at excitation fluence of ~0.5nJ/cm^2 (brown line) and ~0.2nJ/cm^2 (cyan line). The wavelength of the excitation laser was 475 nm. The lifetime shows no dependence on the intensity of the excitation light.

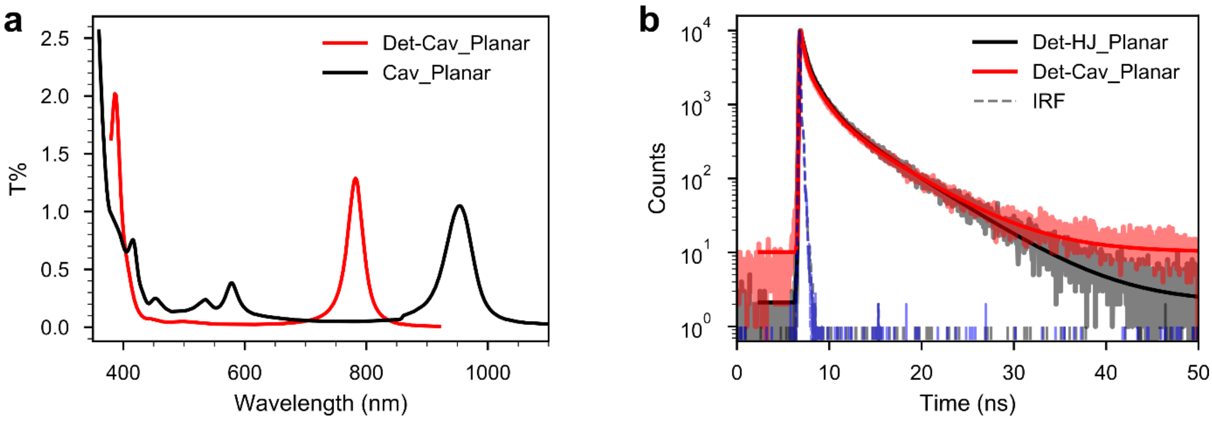

Supplementary Figure 7: a The transmission of the cavity at resonance (Cav_Planar) and far detuned cavity (Det-Cav_Planar). The detuning is achieved by reducing the thickness of DPA from 163 nm to 100 nm (other layers are kept the same). In the detuned cavity, there is no coupling between the cavity and excitons. **b** PL decay at 720 nm from the detuned planar heterojunction in and outside a cavity. The lifetime keeps the same in and outside the cavity taken the baseline difference into account. The higher baseline in Det-Cav_Planar is caused by accumulation of detector noise during the longer measurement time, as the signal from the cavity is weak.

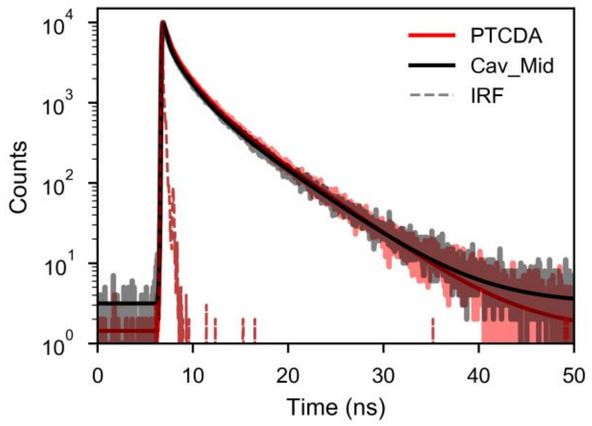

Supplementary Figure 8: PL decay at 720 nm from the Cav_Mid (with a 20-nm PVA layer between DPA and PTCDA) and a PTCDA film (Glass/PTCDA/TmpyTB). The lifetime of the two samples are the same, indicating that the metal mirrors has no affect the lifetime of the PTCDA exciton.

Supplementary Note 4: Calculation of the exciton diffusion length

It is generally known that excimeric states in organic thin films can act as energy traps for singlet exciton transport, resulting in much smaller diffusion lengths than expected. In PTCDA thin films, the energy relaxation from the Frenkel states to the low energy excimeric states is ultrafast (400-500 fs) compared to the typical hopping rates (10^3 to 10^6 fs).[9] The energy of the excimer and polariton are similar in our system; an exchange of energy between these states is therefore expected. Thus, we can use the exciton diffusion model based on decay at 720 nm (from the excimeric state) to determine the relevant diffusion constant and length. The PL decay dynamics were modeled by calculating the number and distribution of the excitations in the film *n(x, t)* according to the 1-D diffusion equation[19,20]:

$$\frac{\partial n(x,t)}{\partial t} = D\frac{\partial^2 n(x,t)}{\partial x^2} - kn(x,t) \qquad (4)$$

where D is the diffusion coefficient, and k is the PL decay rate of the sample without a quenching film. The exciton diffusion model assumes that the unquenched PL decay is mono-exponential. The PL of PTCDA films doesn't follow a single exponential decay due to strong interaction between molecules and the disorder of molecular arrangement. However, in our system, the main effect of the quenching film (DPA) on the decay of PTCDA occurs at early times. The long emission tail almost keeps the same with and without DPA (Figure 3). Therefore, we use k calculated from $k = 1/\tau_e$, where τ_e is the time taken for the PL to fall to 1/e of its initial value for a PTCDA film without a quenching layer. In the diffusion model, the effect of the quenching layer was included by assuming that all excitons which reach the interface are quenched with unit efficiency ($n(L, t)=0$, where x=0 at the TmPyPB/PTCDA interface and L is the thickness of the PTCDA film). As shown in Supplementary Figure 9, the initial distribution of excitons in the film as well as in cavities were obtained from transfer matrix calculations based on the respective electric field distribution (Supplementary Figure 5).[21] The initial exciton distribution in each sample was normalized relative to the exciton number at the TmPyPB/PTCDA interface *(L=0, t=0)*. The diffusion coefficient was obtained by fitting the data using a least squares method. The diffusion equation was numerically solved using a forward Euler scheme and the number of excitons were integrated across the entire film in order to determine the total PL intensity at time *t*. The time range in the emission decay used for the fitting was from 0 to τ_e, and the average diffusion length L_d is given by $L_d = \sqrt{D\tau_e}$. The calculated values of D in the planar HJ film and the cavity are 2.0×10^{-4} cm^2s^{-1} and 1.7×10^{-3} cm^2s^{-1}, respectively, and the exciton diffusion length is 5.4 nm in HJ_planar and 16.1 nm in Cav_Planar. The exciton diffusion length was calculated based on the assumption that the exciton dissociation efficiency at the DPA/PTCDA interface is unity and no exciton exists at the interface. The exciton dissociation rate is ultrafast compared to the rate of exciton decay as discussed in Supplementary Note 1, supporting this assumption. Strictly speaking, we obtained a minimum value as for the diffusion coefficient, since with a finite quenching rate a larger diffusion coefficient would be required to achieve the same amount quenching.

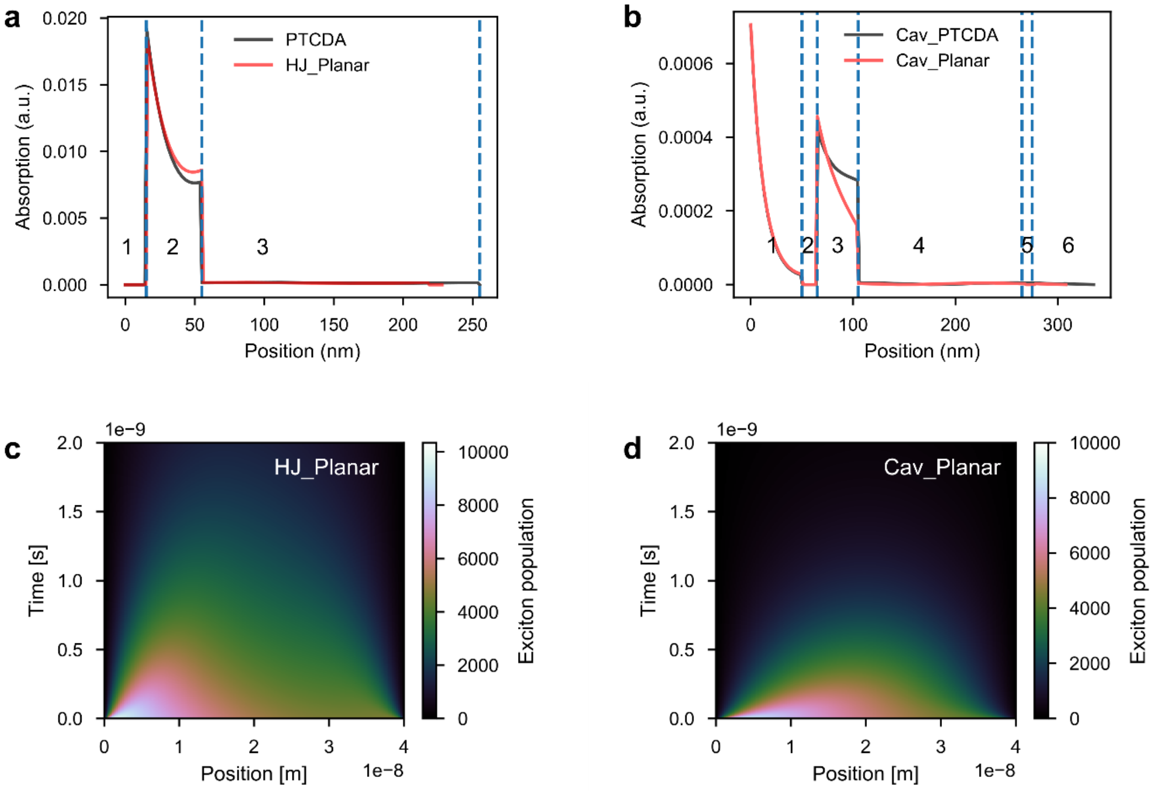

Supplementary Figure 9: a The absorption profile at 475 nm in the planar HJ film (a) and cavity (b) as calculated with the transfer matrix approach. The vertical blue line indicate the interface between different layers. The sample structure is 15-nm TmPyPB (1), 40-nm PTCDA (2) and 163-nm DPA or 185-nm PS (3). **b** The absorption profile at 475 nm in the cavity. The sample structure is 50-nm Ag (1), 15-nm TmPyPB (2), 40-nm PTCDA (3), 163-nm DPA or 185-nm PS (4), 10-nm mCP (5) and 30-nm Ag (6). **c, d** The exciton diffusion in the PTCDA layer in the planar heterojunction film (c) and cavity (d) as calculated based on the one-dimension exciton diffusion model. The interface between TmPyPB and PTCDA is defined as 0 nm in the plot. The initial exciton population is set as 10^4.

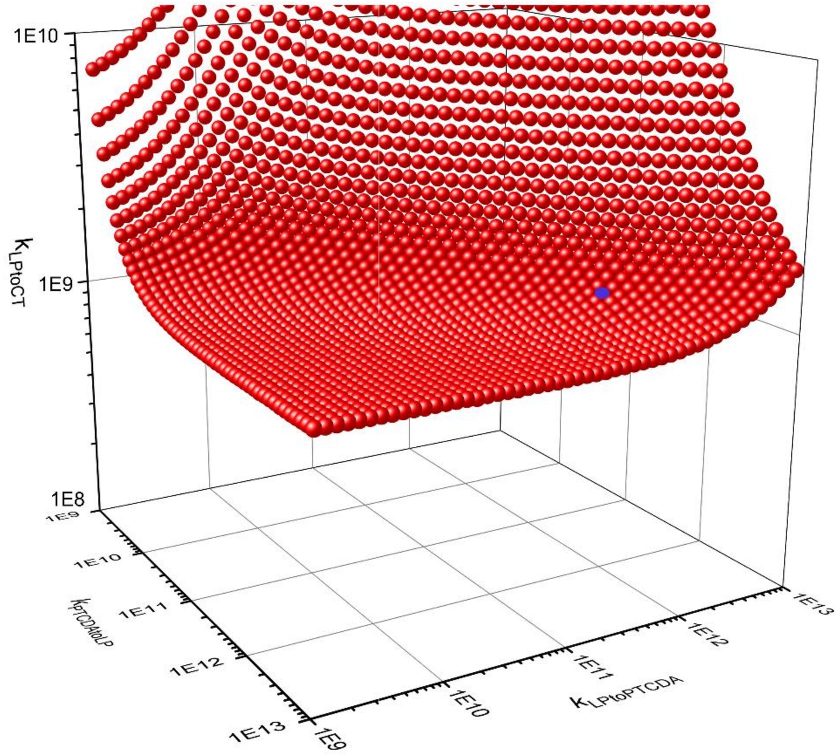

Supplementary Figure 10: Showing the value of k_{LPtoCT} as a function of set values of $k_{\mathrm{PTCDAtoLP}}$ and $k_{\mathrm{LPtoPTCDA}}$ in the range of 10^9 to 10^{13} s^{-1}. Irrespective of values for $k_{\mathrm{LPtoPTCDA}}$ and $k_{\mathrm{PTCDAtoLP}}$, the obtained k_{LPtoCT} is much larger as compared to k_{CT} ($1.7 \cdot 10^8$ s^{-1}). Furthermore, the stated value of k_{LPtoCT} in the article ($1.74 \cdot 10^9$ s^{-1}, indicated by the blue dot) can be viewed as a conservative estimate considering the calculated values of k_{LPtoCT} within the parameter space.

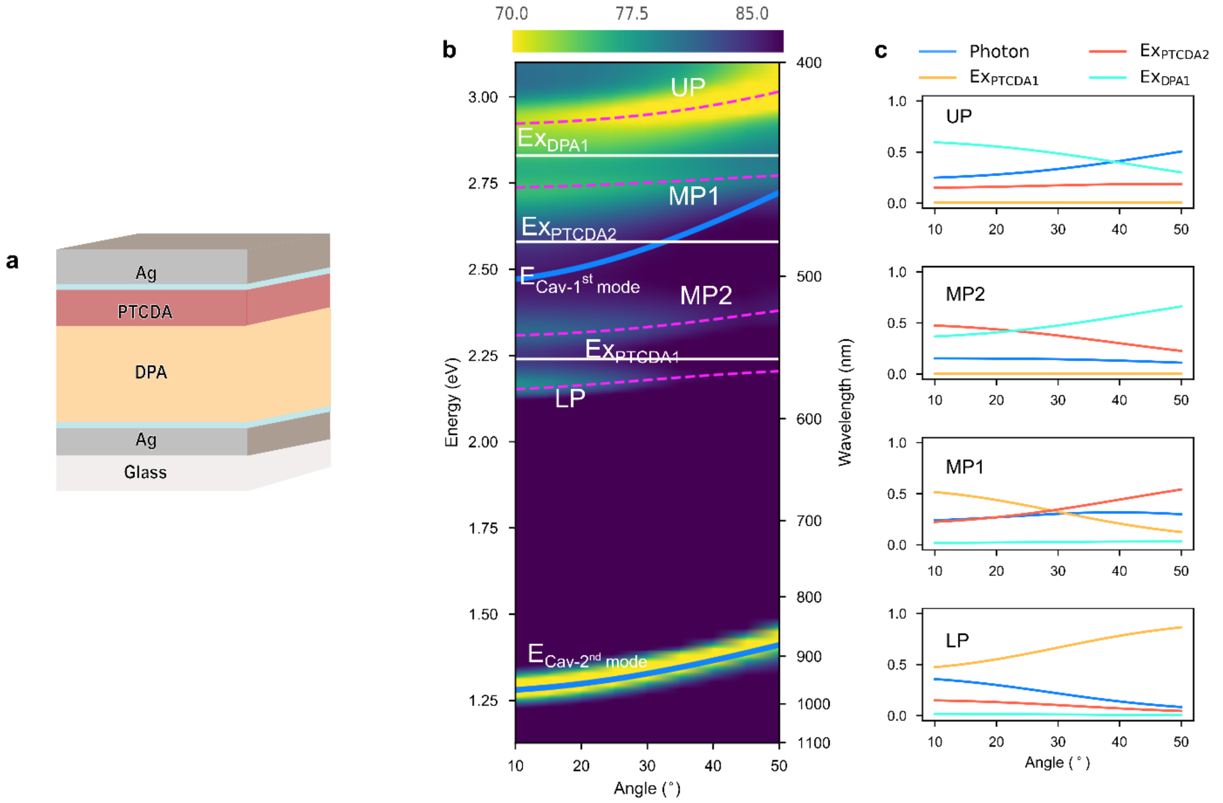

Supplementary Figure 11: a Schematic illustration of the cavity in the study: 30-nm Ag / 10-nm mCP / 163-nm DPA / 40-nm PTCDA / 15-nm / TmPyPB / 50 nm Ag. **b** Plot of the angle-resolved reflectivity (TE mode) and the fitting results from a 4-by-4 coupled oscillator model. **c** The Hopfield coefficients of each polariton.

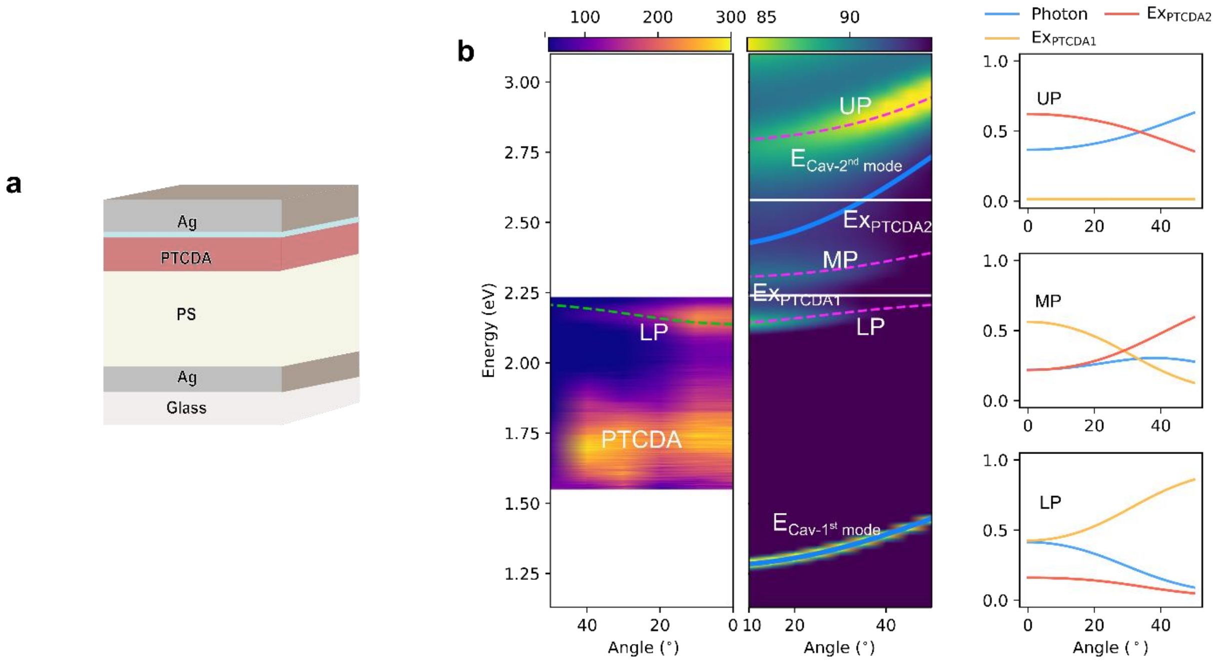

Supplementary Figure 12: **a** Cavity structure of the reference cavity: 30-nm Ag / ~185-nm PS / 40-nm PTCDA / 15-nm TmPyPB / 50-nm Ag **b**. Plot of angle-resolved emission (right) of Cav_PTCDA. PL emission from Cav_PTCDA under the excitation of 475 nm (2.61 eV). The emission from PL shows dispersive behavior with angle increases, while the emission of PTCDA at 1.75 eV is non-dispersive. Angle-resolved reflectivity (TE mode, right) and the fitting results from a 3-by-3 coupled oscillator model. The Hopfield coefficients of each polariton are shown on the far-right side.

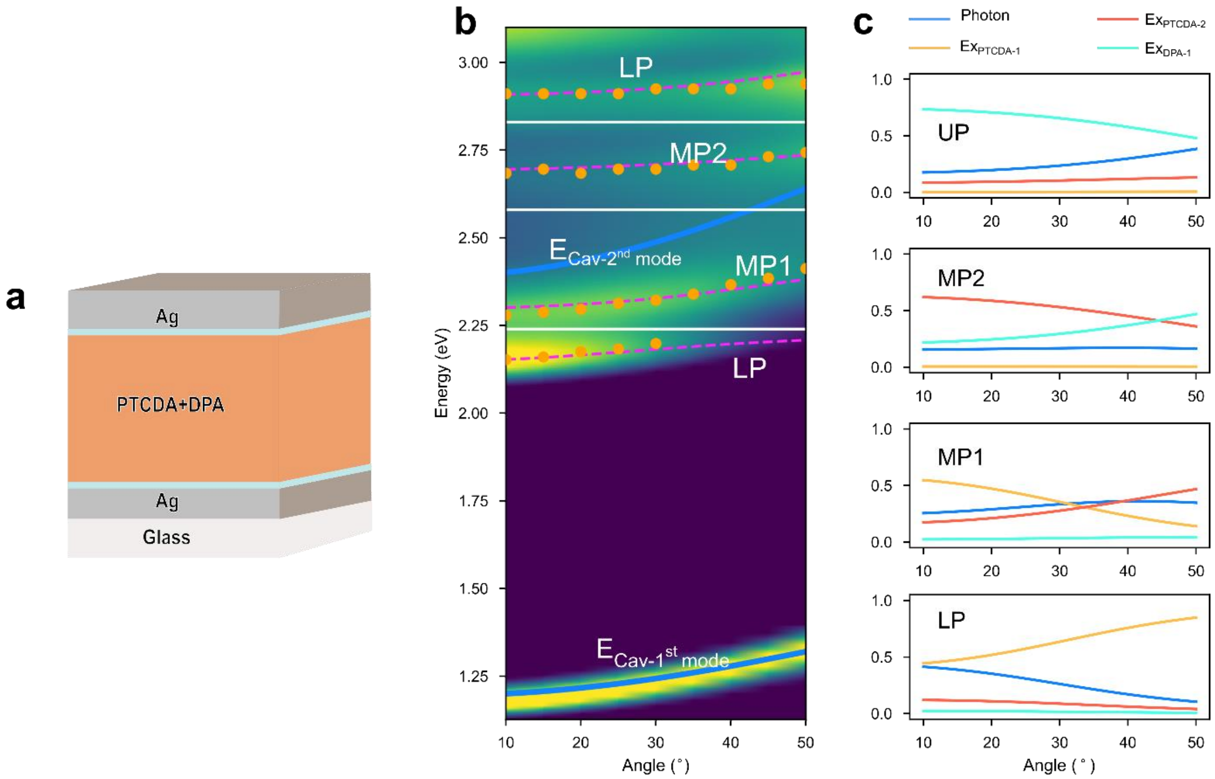

Supplementary Figure 13: **a** Schematic of the bulk heterojunction cavity in the study: 30-nm Ag / 10-nm mCP / 163-nm DPA + 40-nm PTCDA / 15-nm TmPyPB / 50-nm Ag. **b** Plot of the angle-resolved reflectivity (TE mode) and the fitting results from a 4-by-4 coupled oscillator model. **c** The Hopfield coefficients of each polariton.

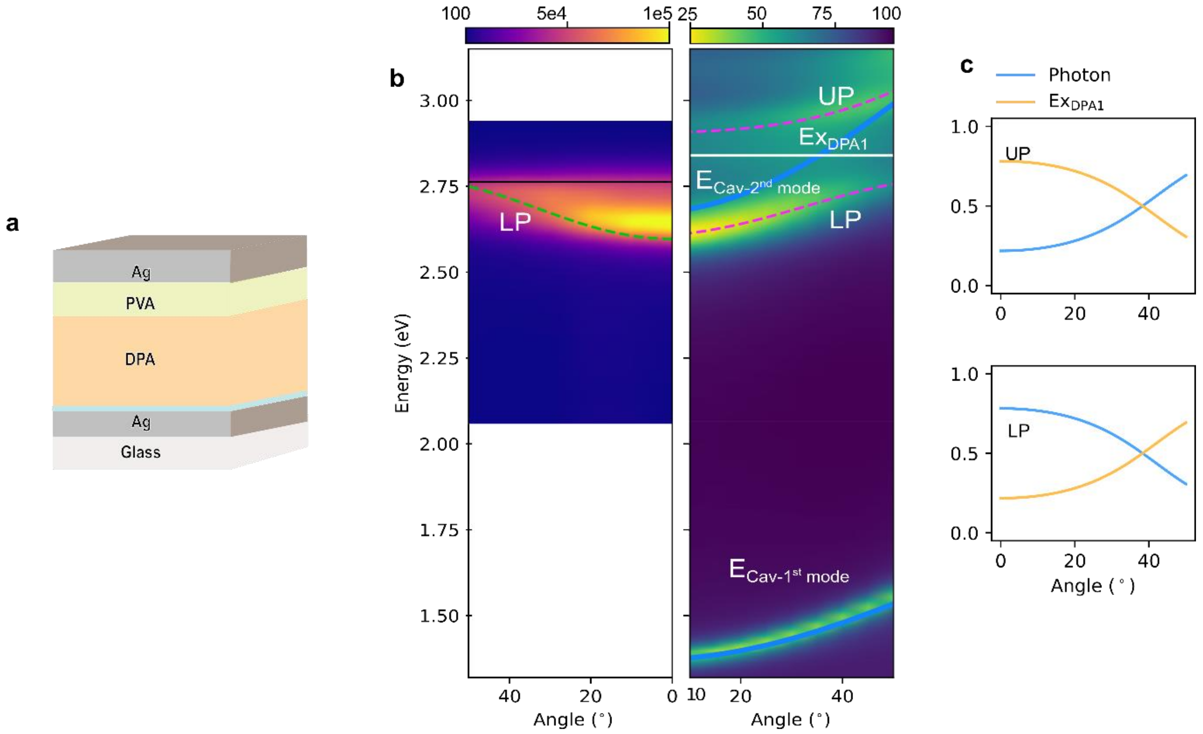

Supplementary Figure 14: **a** Cavity structure of the reference cavity (Cav_DPA): 30-nm Ag / 10-mCP / 163-nm DPA / ~80-nm PVA / 50-nm Ag. **b** Angle-resolved emission (left) of the cavity. PL emission for Cav_DPA probed from various angles under the excitation of 405 nm. The LP moves towards high energy as the probe angle increases. The black line indicates the emission position of DPA molecules. The angle-resolved reflectivity (right, TE mode). The reflection data was fitted by a 2-by-2 coupled oscillator model. **c** The Hopfield coefficients of each polariton.

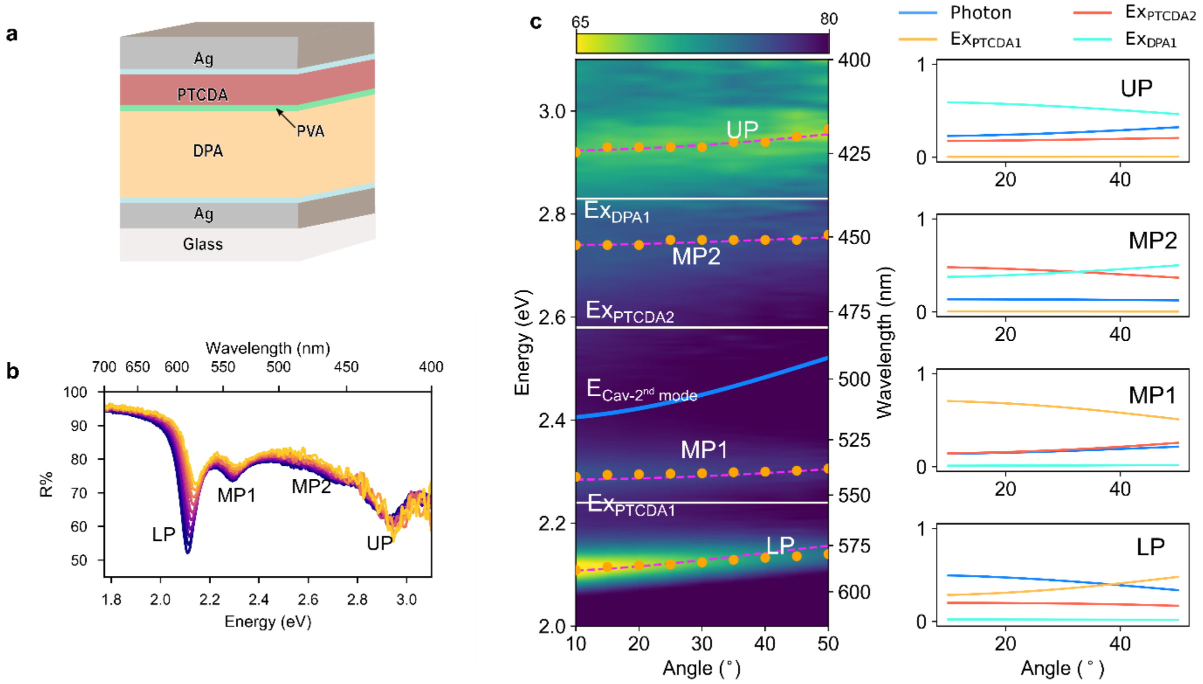

Supplementary Figure 15: **a** Cavity structure of the reference cavity (Cav_Mid): 30-nm Ag / 5-mCP / 143-nm DPA / ~20-nm PVA / 40-nm PTCDA / 15-nm TmPyPB / 30-nm Ag. **b** Angle-resolved reflectivity (TE mode) of the cavity: from 10 to 50 degrees. **c** Plot of angle-resolved reflectivity (TE) and the fitting results from a 4-by-4 coupled oscillator model. The Hopfield coefficients of each polariton are shown on the right side.

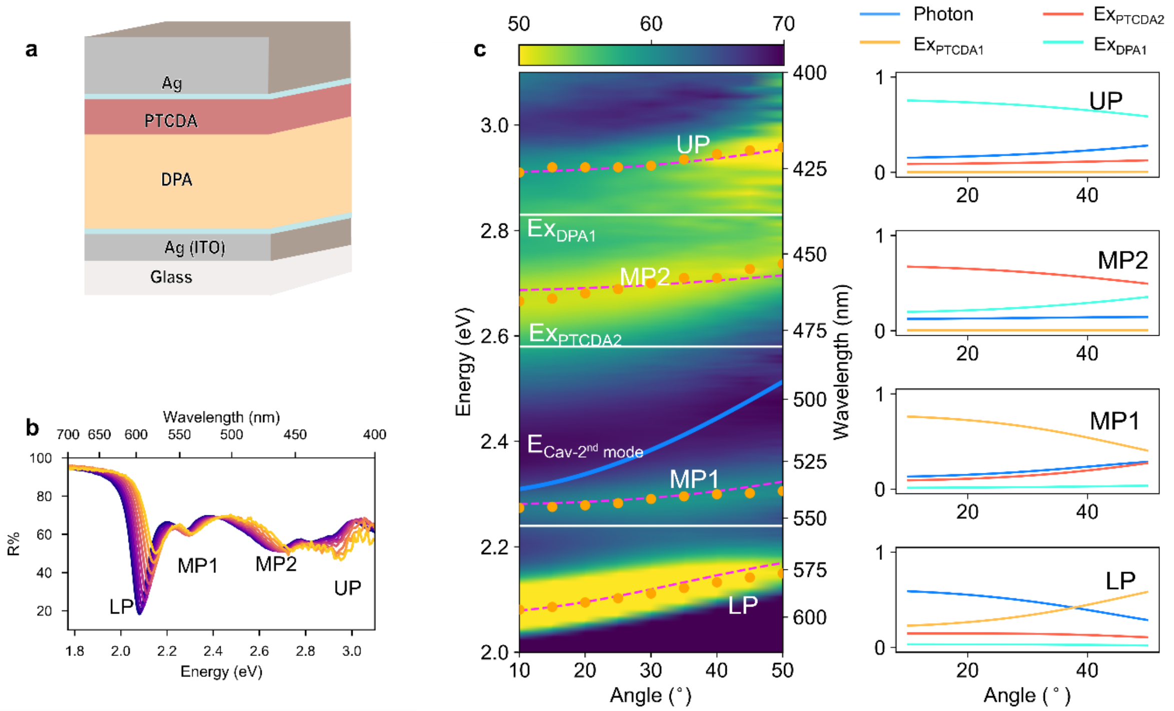

Supplementary Figure 16: **a** The geometry structure of the cavity device and reference device. (20-nm Ag (110-nm ITO) / 10-mCP / 173-nm DPA / 45-nm PTCDA / 10-nm TmPyPB / 120-nm Ag). The only difference is the bottom electrode. It is 20nm-Ag for Cavity device and 110-nm ITO for reference device. **b** Angle-resolved reflectivity (TE mode) of the cavity device: from 10 to 50 degrees. **c** Plot of angle-resolved reflectivity (TE) and the fitting results from a 4-by-4 coupled oscillator model. The Hopfield coefficients of each polariton are shown on the right side.

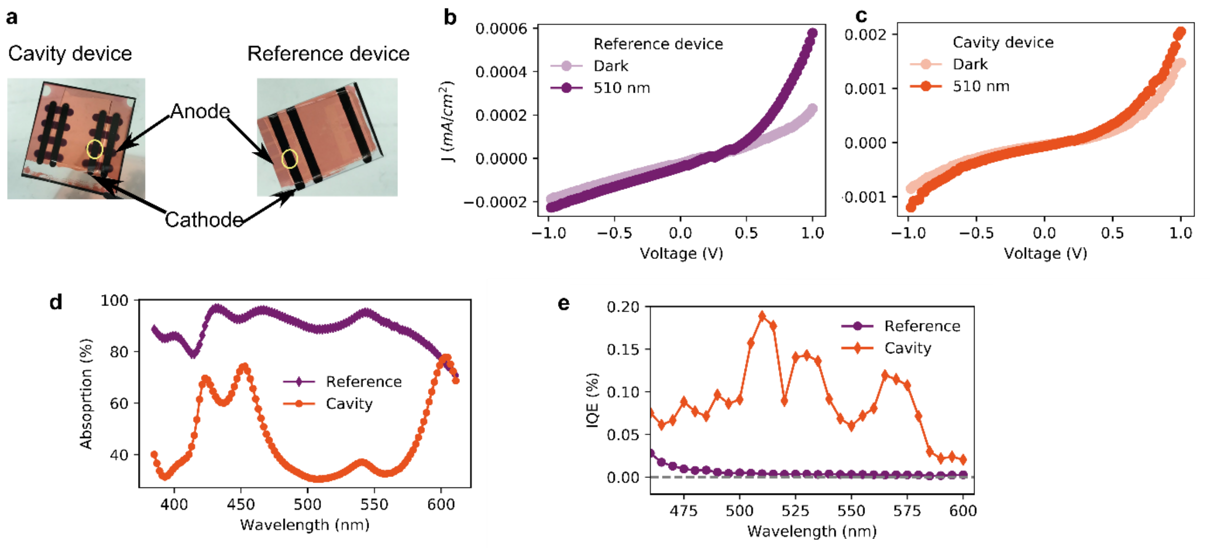

Supplementary Figure 17: **a** Photos of the cavity and reference photodiode. The device area, highlighted by the yellow circle in the figure is defined by the cross area of bottom anode and top cathode. The active junction size amounts to 0.2 cm × 0.15 cm. The only difference between cavity and reference device is the bottom anode: 20 nm Ag for Cavity and 110 nm ITO for Reference device. **b** and **c** Current-voltage (J-V) characteristics for reference (b) and cavity devices (c) under dark and illumination. The light intensity of 510 nm illumination is 2.1mW/cm^2. **d** The absorption of the devices from TMM calculation. **e** IQE of the cavity and reference devices.

Supplementary Table 1. The structures of film and cavity samples in the study and the corresponding abbreviations.

Abbreviation	Sample structure
PTCDA	Glass / ~185-nm PS / 40-nm PTCDA / 15-nm TmPyPB
DPA	Glass /10-nm mCP / 163-nm DPA / ~80-nm PVA
HJ_Planar	Glass / 10-nm mCP / 163-nm DPA / 40-nm PTCDA / 15-nm TmPyPB
HJ_Bulk	Glass / 10-nm mCP / 163-nm DPA + 40-nm PTCDA / 15-nm TmPyPB
Det-HJ_Planar	Glass / 10-nm mCP / 100-nm DPA / 40-nm PTCDA / 15-nm TmPyPB
Cav_PTCDA	Glass / 30-nm Ag / ~185-nm PS / 40-nm PTCDA / 15-nm TmPyPB / 50-nm Ag
Cav_Planar	Glass / 30-nm Ag / 10-nm mCP / 163-nm DPA / 40-nm PTCDA / 15-nm TmPyPB / 50-nm Ag
Cav_Bulk	Glass / 30-nm Ag /10-nm mCP / 163-nm DPA+40-nm PTCDA / 15-nm TmPyPB/ 50-nm Ag
Det-Cav_Planar	Glass / 30-nm Ag / 10-nm mCP / 100-nm DPA / 40-nm PTCDA / 15-nm TmPyPB / 50-nm Ag
Cav_DPA	Glass / 30-nm Ag /10-nm mCP / 163-nm DPA / ~80-nm PVA / 50-nm Ag
Cav_Mid	Glass / 30-nm Ag / 10-mCP / 143-nm DPA / ~20-nm PVA / 40-nm PTCDA / 15-nm TmPyPB / 30-nm Ag
Cavity device	Glass / 20-nm Ag/ 5-mCP / 173-nm DPA / 45-nm PTCDA / 10-nm TmPyPB / 120-nm Ag
Reference device	Glass / 110-nm ITO/ 5-mCP / 173-nm DPA / 45-nm PTCDA / 10-nm TmPyPB / 120-nm Ag

Supplementary Table 2. The Rabi splittings and effective refractive indices of the cavities in the study obtained from the fitting with a coupled oscillator model. The FWHM of the excitons in the system are 0.17 eV (Ex_{PTCDA1}), 0.36 eV (Ex_{PTCDA2}) and 0.15 eV (Ex_{DPA1}). The values to compare are Ex_{PTCDA1} with $\hbar\Omega_1$, Ex_{PTCDA2} with $\hbar\Omega_2$, and Ex_{PTCDA3} with $\hbar\Omega_3$. The quality factor of the cavities are roughly 20-30, based on the lambda/2 mode. The error bars of the fitting parameters here are from the leastsq method. Other factors that may cause errors including inhomogeneity of the cavity, and peak positions of polaritons are considered negligible compared to the errors from the fitting.

	$\hbar\Omega_1$ (eV)	$\hbar\Omega_2$ (eV)	$\hbar\Omega_3$ (eV)	n_{eff}
Cav_PTCDA	0.20 ± 0.04	0.54 ± 0.01	---	1.65± 0.44
Cav_Planar	0.20 ± 0.09	0.51 ± 0.04	0.29 ± 0.02	1.79 ± 0.17
Cav_Bulk	0.15 ± 0.05[a]	0.42 ± 0.03	0.29 ± 0.03	1.61 ± 0.16
Cav_PVA	--	--	0.24 ± 0.01	1.82 ± 0.04
Cav_Mid	0.20 ± 0.09	0.60 ± 0.04	0.30± 0.03	2.5± 0.36
Cavity Device	0.20± 0.07	0.50 ± 0.04	0.36± 0.05	1.9 ± 0.17

[a]The Ex_{PTCDA1} depends much on the aggregation of PTCDA molecules, as it arises from the mixed Frenkel and charge-transfer states. In the bulk heterojunction, PTCDA molecules aggregate differently compared to that of a pure PTCDA film. The FWHM of the Ex_{PTCDA1} is 0.13 eV based on the absorption spectrum, which is smaller than the obtained Rabi splitting ($\hbar\Omega_1 = 0.15\ eV$).

Supplementary Table 3. The Hopfield coefficients at $k_{//}$=0 (angle=0 ^{o}) of each polariton in planar heterojunction (Cav_Planar) in the strong coupling regime.

	Photon	Ex_{PTCDA1}	Ex_{PTCDA2}	Ex_{DPA1}
LP	0.38	0.43	0.17	~ 0.02
MP1	0.21	0.56	0.21	~ 0.02
MP2	0.14	< 0.01	0.42	0.42
UP	0.26	< 0.01	0.17	0.55

Supplementary Table 4. The Hopfield coefficients at $k_{//}$=0 (angle=0 º) of each polariton of the reference cavity (Cav_PTCDA) in the strong coupling regime.

	Photon	Ex_{PTCDA1}	Ex_{PTCDA2}
LP	0.41	0.44	0.15
MP	0.23	0.54	0.23
UP	0.35	0.02	0.63

Supplementary Table 5. The Hopfield coefficients at $k_{//}$=0 (angle=0º) of each polariton in the bulk heterojunction in the strong coupling regime.

	Photon	Ex_{PTCDA1}	Ex_{PTCDA2}	Ex_{DPA1}
LP	0.41	0.44	0.12	~ 0.02
MP1	0.25	0.56	0.17	~ 0.02
MP2	0.15	< 0.01	0.63	0.22
UP	0.17	< 0.01	0.1	0.73

Supplementary Table 6. The Hopfield coefficients at $k_{//}$=0 (angle=0º) of each polariton in the DPA reference cavity in the strong coupling regime.

	Photon	Ex_{DPA1}
LP	0.79	0.21
UP	0.21	0.79

Supplementary Table 7. The Hopfield coefficients at $k_{//}$=0 (angle=0º) of each polariton in Cav_Mid (30-nm Ag / 10-mCP / 143-nm DPA / ~20-nm PVA / 40-nm PTCDA / 15-nm TmPyPB / 30-nm Ag.).

	Photon	Ex_{PTCDA1}	Ex_{PTCDA2}	Ex_{DPA1}
LP	0.49	0.28	0.20	~ 0.02
MP1	0.14	0.70	0.14	~ 0.01
MP2	0.14	< 0.01	0.48	0.37
UP	0.23	< 0.01	0.17	0.59

Supplementary Table 8. The Hopfield coefficients at $k_{//}$=0 (angle=0º) of each polariton in cavity device (20-nm Ag / 10-mCP / 173-nm DPA / 45-nm PTCDA / 10-nm TmPyPB / 120-nm Ag.).

	Photon	Ex_{PTCDA1}	Ex_{PTCDA2}	Ex_{DPA1}
LP	0.59	0.23	0.15	~ 0.03
MP1	0.13	0.76	0.09	~ 0.01
MP2	0.12	< 0.01	0.67	0.19
UP	0.15	< 0.01	0.08	0.75

Supplementary Table 9. The lifetime of DPA and the rate of energy transfer of the heterojunction films in and outside cavities.

	Lifetime of DPA (ns)[a]	Rate of energy transfer from DPA to PTCDA ($\times 10^9$ s^{-1})[b]
DPA	$0.83 \pm 0.2\times10^{-3}$	
Cav_DPA	$1.64 \pm 0.2\times10^{-3}$	
HJ_Planar	$0.56 \pm 0.2\times10^{-3}$	0.58 ± 0.001
Cav_Planar	$0.50 \pm 0.3\times10^{-3}$	1.39 ± 0.001
HJ_Bulk	$0.34 \pm 0.3\times10^{-3}$	1.73 ± 0.003
Cav_Bulk	0.15 ± 0.02	6.06 ± 0.89

[a]The lifetimes of DPA of all films and cavities are probed at 448nm and are calculated based on a three-exponential model convoluted with the instrument response function (IRF): $\tau_{avg} = \sum_i \tau_i{}^2 B_i/(\tau_i B_i)$, where, B_i is the amplitude of the corresponding component. The error bars of the average lifetimes are obtained from the deconvolution fitting of the decay. [b]For the heterojunction film outside the cavity, the rate of energy transfer is calculated from: $k_{ET} = 1/\tau_{HJ} - 1/\tau_{DPA}$. Inside the cavities, the rate of energy transfer is calculated from: $k_{ET}^{Cav} = 1/\tau_{HJ}^{Cav} - 1/\tau_{DPA}^{Cav}$. The error bars of the rate of charge transfer are calculated based on the propagation of errors from the corresponding lifetimes.

Supplementary References

1 Scully, S. R., Armstrong, P. B., Edder, C., Fréchet, J. M. J. & McGehee, M. D. Long-range resonant energy transfer for enhanced exciton harvesting for organic solar cells. *Adv. Mater.* **19**, 2961-2966 (2007).

2 Főrster, T. 10th spiers memorial lecture. Transfer mechanisms of electronic excitation. *Discuss. Faraday Soc.* **27**, 7-17 (1959).

3 Lakowicz, J. R. *Principles of fluorescence spectroscopy*. (Springer Science & Business Media, 2013).

4 Marcus, R. A. & Sutin, N. Electron transfers in chemistry and biology. *Biochimica et Biophysica Acta (BBA) - Reviews on Bioenergetics* **811**, 265-322 (1985).

5 Rand, B. P., Burk, D. P. & Forrest, S. R. Offset energies at organic semiconductor heterojunctions and their influence on the open-circuit voltage of thin-film solar cells. *Phys. Rev. B* **75**, 115327 (2007).

6 Möbus, M., Karl, N. & Kobayashi, T. Structure of perylene-tetracarboxylic-dianhydride thin films on alkali halide crystal substrates. *J. Cryst. Growth* **116**, 495-504 (1992).

7 Forrest, S. R., Kaplan, M. L. & Schmidt, P. H. Organic ‐ on ‐ inorganic semiconductor contact barrier diodes. Ii. Dependence on organic film and metal contact properties. *J. Appl. Phys.* **56**, 543-551 (1984).

8 Heutz, S., Ferguson, A. J., Rumbles, G. & Jones, T. S. Morphology, structure and photophysics of thin films of perylene-3,4,9,10-tetracarboxylic dianhydride. *Org. Electron.* **3**, 119-127 (2002).

9 Settels, V. *et al.* Identification of ultrafast relaxation processes as a major reason for inefficient exciton diffusion in perylene-based organic semiconductors. *J. Am. Chem. Soc.* **136**, 9327-9337 (2014).

10 Bellinger, D., Pflaum, J., Brüning, C., Engel, V. & Engels, B. The electronic character of ptcda thin films in comparison to other perylene-based organic semi-conductors: Ab initio-, td-dft and semi-empirical computations of the opto-electronic properties of large aggregates. *PCCP* **19**, 2434-2448 (2017).

11 So, F. F. & Forrest, S. R. Evidence for exciton confinement in crystalline organic multiple quantum wells. *Phys. Rev. Lett.* **66**, 2649-2652 (1991).

12 Hoffmann, M. & Soos, Z. G. Optical absorption spectra of the holstein molecular crystal for weak and intermediate electronic coupling. *Phys. Rev. B* **66**, 024305 (2002).

13 Hennessy, M. H., Pascal, R. A. & Soos, Z. G. Vibronic structure of frenkel and charge-transfer excitons in ptcda. *Mol. Cryst. Liq. Cryst. Sci. Technol., Sect. A* **355**, 41-63 (2001).

14 Scholz, R., Kobitski, A. Y., Vragović, I., Wagner, H. P. & Zahn, D. R. T. Time-resolved photoluminescence in α-ptcda single crystals: Evidence for recombination via frenkel excitons, charge transfer states, and excimers. *Org. Electron.* **5**, 99-105 (2004).

15 Kobitski, A. Y. *et al.* Time-resolved photoluminescence study of excitons in thin ptcda films at various temperatures. *Appl. Surf. Sci.* **212-213**, 428-432 (2003).

16 Stallberg, K., Namgalies, A. & Höfer, U. Photoluminescence study of the exciton dynamics at ptcda/noble-metal interfaces. *Phys. Rev. B* **99**, 125410 (2019).

17 Ferguson, A. J. & Jones, T. S. Photophysics of ptcda and me-ptcdi thin films: Effects of growth temperature. *J. Phys. Chem. B* **110**, 6891-6898 (2006).

18 Scully, S. R. & McGehee, M. D. Effects of optical interference and energy transfer on exciton diffusion length measurements in organic semiconductors. *J. Appl. Phys.* **100**, 034907 (2006).

19 Shaw, P. E., Ruseckas, A. & Samuel, I. D. W. Exciton diffusion measurements in poly(3-hexylthiophene). *Adv. Mater.* **20**, 3516-3520 (2008).

20 Stranks, S. D. *et al.* Electron-hole diffusion lengths exceeding 1 micrometer in an organometal trihalide perovskite absorber. *Science* **342**, 341-344 (2013).

21 Furman, S. A. & Tikhonravov, A. *Basics of optics of multilayer systems*. (Atlantica Séguier Frontieres, 1992).
